# Oncogenic composite mutations can be predicted by co‐mutations and their chromosomal location

**DOI:** 10.1002/1878-0261.13636

**Published:** 2024-05-16

**Authors:** Asli Küçükosmanoglu, Carolien L. van der Borden, Lisanne E. A. de Boer, Roel Verhaak, David Noske, Tom Wurdinger, Teodora Radonic, Bart A. Westerman

**Affiliations:** ^1^ Department of Neurosurgery, Brain Tumor Center Amsterdam Amsterdam University Medical Center, Cancer Center Amsterdam The Netherlands; ^2^ Department of Computational Biology The Jackson Laboratory for Genomic Medicine Farmington CT USA; ^3^ Department of Pathology Amsterdam University Medical Center, Cancer Center Amsterdam The Netherlands

**Keywords:** composite mutations, genetic heterogeneity, parallel evolution, predictive model, therapy resistance

## Abstract

Genetic heterogeneity in tumors can show a remarkable selectivity when two or more independent genetic events occur in the same gene. This phenomenon, called composite mutation, points toward a selective pressure, which frequently causes therapy resistance to mutation‐specific drugs. Since composite mutations have been described to occur in sub‐clonal populations, they are not always captured through biopsy sampling. Here, we provide a proof of concept to predict composite mutations to anticipate which patients might be at risk for sub‐clonally driven therapy resistance. We found that composite mutations occur in 5% of cancer patients, mostly affecting the *PIK3CA*, *EGFR*, *BRAF*, and *KRAS* genes, which are common precision medicine targets. Furthermore, we found a strong and significant relationship between the frequencies of composite mutations with commonly co‐occurring mutations in a non‐composite context. We also found that co‐mutations are significantly enriched on the same chromosome. These observations were independently confirmed using cell line data. Finally, we show the feasibility of predicting compositive mutations based on their co‐mutations (AUC 0.62, 0.81, 0.82, and 0.91 for *EGFR*, *PIK3CA*, *KRAS*, and *BRAF*, respectively). This prediction model could help to stratify patients who are at risk of developing therapy resistance‐causing mutations.

## Graphical abstract

Composite mutations, found in 5% of cancer patients, frequently drive therapy resistance, indicating parallel tumor evolution alongside specific co‐mutations. Identifying these co‐mutations reveals their common occurrence in the same genomic region, potentially aiding in detecting residual therapy‐resistant composite mutations. Our approach may enable targeting such mutations, even when undetected in bulk or liquid biopsies, benefiting patient selection for tailored treatments.

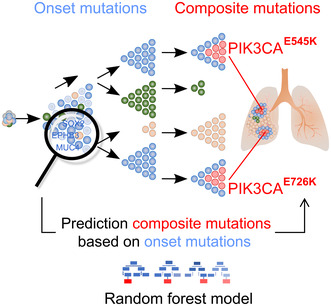

**Abbreviations**
AUC: area under the curve
*BRAF*
: B‐Raf proto‐oncogene, serine/threonine kinase
CCLE database: Cancer Cell Line Encyclopedia database
*CDH1*
: cadherin‐1 (E‐Cadherin)
*CDKN2A*
: cyclin‐dependent kinase inhibitor 2A
CNV: copy number variation
COSMIC: catalog of somatic mutations in cancer
CRISPR: clustered regularly interspaced short palindromic repeats (a gene editing technology)
DNA: deoxyribonucleic acid
*EEF1A1*
: eukaryotic translation elongation factor 1 alpha 1
*EGFR*
: epidermal growth factor receptor
*ERBB2*
: receptor tyrosine‐protein kinase ErbB‐2 (also known as HER2)
*ERBB3*
: receptor tyrosine‐protein kinase ErbB‐3 (also known as HER3)
FN: false negative
FP: false positive
FPR: false‐positive rate
GEM: The Generic Enrichment Map
GRCh38: genome reference consortium human build 38 (Organism: *Homo sapiens*)
*HRAS*
: Harvey rat sarcoma viral oncogene homolog
*KIT*
: KIT proto‐oncogene receptor tyrosine kinase
*KRAS*
: Kirsten rat sarcoma viral oncogene homolog
mRNA: messenger ribonucleic acid
MSK: Memorial Sloan Kettering Cancer Center
*MTOR*
: mechanistic target of rapamycin
*NRAS*
: neuroblastoma RAS viral oncogene homolog
*PDGFRA*
: platelet‐derived growth factor receptor alpha
*PIK3CA*
: phosphatidylinositol‐4,5‐bisphosphate 3‐kinase catalytic subunit alpha
*PTEN*
: phosphatase and tensin homolog
ROC: receiver operator curve performance representation
*SMAD4*
: SMAD family member 4
SRC: survival, regression, and classification
TCGA: The Cancer Genome Atlas
TKI: tyrosine kinase inhibitor
TN: true negative
*TP53*
: tumor protein p53
TPR: true‐positive rate
UMC: University Medical Center
UV: ultraviolet
*VHL*
: Von Hippel–Lindau tumor suppressor
VUMC: Vrije Universiteit Medical Center

## 1 Introduction

Tumor formation is a complex genetic and clonal evolution process that takes place before a tumor is detected [1, 2, 3]. This continued spatiotemporal selection process can accumulate considerable levels of intratumoral heterogeneity involved in therapy resistance. For example, lung cancer patients with *EGFR*‐activated tumors commonly show therapy resistance through additional mutations in the *EGFR* gene, notably the T790M mutation, now targeted by third‐generation TKI inhibitors such as Osimertinib [4, 5]. Interestingly, this resistance‐causing mutation is already present in 17% of untreated patients [6]. Similarly, in patients with *BRAF*‐activated tumors, therapy resistance against dabrafenib or vemurafenib is observed through amplification of the wild‐type allele [7]. Targeted therapy against the *KRAS* V12C mutation has recently become available, and although clinical data are still limited [8], therapy resistance through recurrent mutations could also play a role here as well. Therefore, intratumoral heterogeneity is associated with poorer clinical outcomes and is considered one of the major drivers of therapy resistance [9, 10, 11].

Intratumoral heterogeneity is considered a consequence of dynamics that unfold depending on local cues and selection pressures during clonal evolution [11, 12, 13, 14, 15]. The intratumoral heterogeneity of driver mutations is considered to be driven by stochastic evolution (Big Bang Evolution [16, 17]), where the tumor gradually obtains mutations, leading to an accumulation of heterogenous tumor‐driving lesions in subsequent clonal populations. Alternatively, clonal populations of cells can also show a spatial drift during tumor formation, leading to clonally derived areas containing different sets of somatic mutations with a shared ancestor (multiverse evolution [17]). Although this suggests that the complexity of mutations versus cellular phenotypic states might be inexhaustible, genetic evolution paths turn out to be rather conserved and linked to tumor types. This suggests that the epigenetic programming of the cell of origin in combination with the tissue context (microenvironmental) cues defines the onset and progression path of the tumor [18].

Intratumoral heterogeneity is commonly associated with mutations but also with chromosomal gains and losses, indicated by copy number variations (CNVs). CNVs affect the expression levels of genes, especially when a certain oncogene is amplified or when a tumor suppressor gene has a deep deletion. Furthermore, in many solid tumor types, rearrangements of chromosomes occur. An extreme case of these rearrangements is called chromothripsis [19] (from the Greek for “chromosome” (*chromo*) and “shattering into pieces” (*thripsis*)), where one or more chromosomes have been rearranged from hundreds of chromosomal fragments. Multiple myeloma and neuroblastoma are tumor types that commonly show chromothripsis, which is associated with a poor clinical outcome [20].

Cancers such as lung or skin cancer (melanoma), which frequently arise as a result of exposure to exogenous mutagens, such as UV light and tobacco carcinogens, have relatively high numbers of mutations. These mutations result from malfunction of DNA replication which results commonly in a purine for a purine (A to G or reverse) or a pyrimidine for a pyrimidine (C to T or reverse) exchange. Given the high mutation rates in these tumor types, they can harbor up to 8000 heterogeneous coding mutations between primary and metastatic or recurrence sites [21]. Most tumors, however, show between 10 and 100 heterogeneous mutations [10]. Additional analyses of single cells within tumors have confirmed that tumors consist of heterogeneous populations of cells as observed from differences on a genetic level as well as on the phenotypic (transcriptomic/protein/metabolic) level [22, 23, 24, 25].

**Fig. 1:**
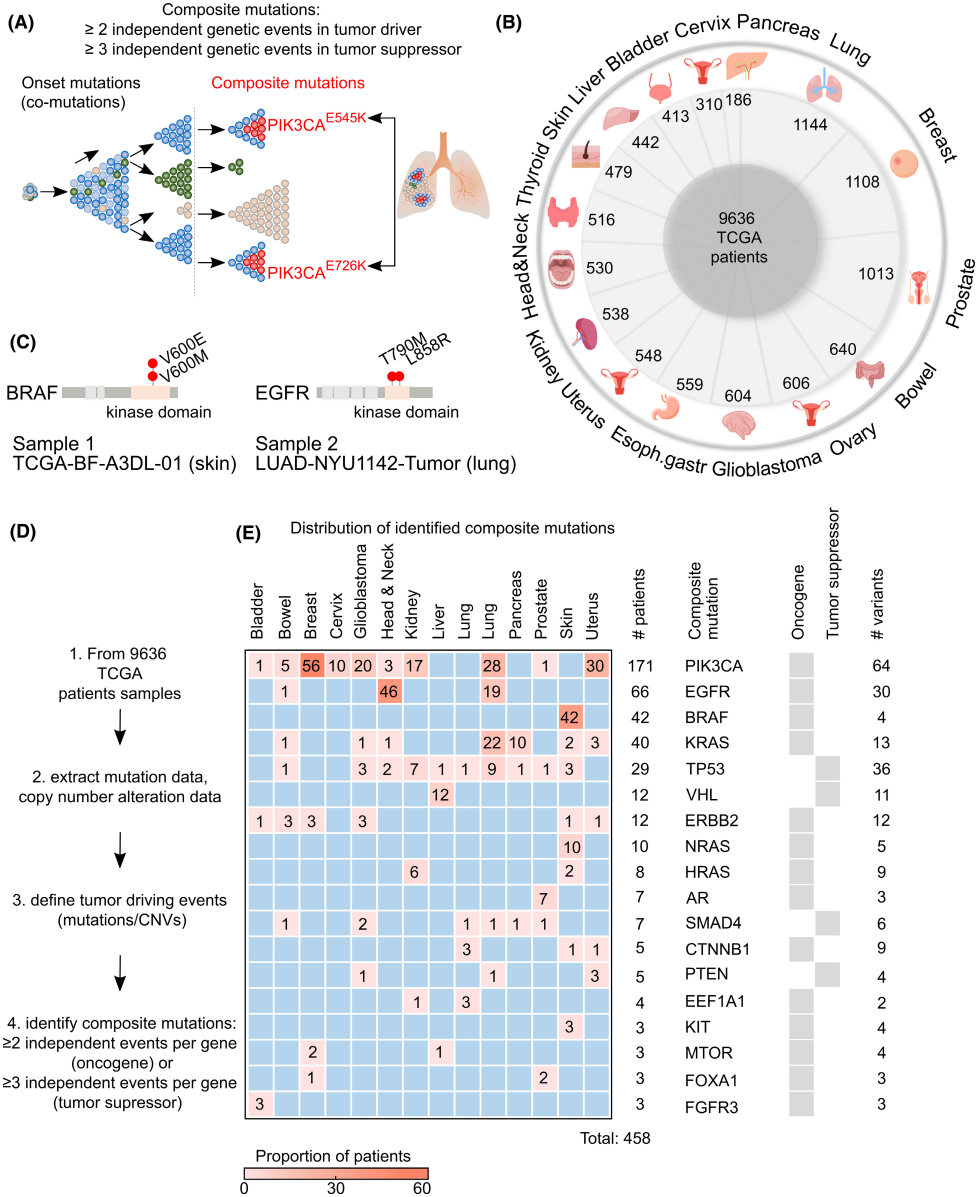
Composite mutations are present in 5% of patients. (A) Cartoon showing the concept of composite mutations derived from the clonal outgrowth in a tumor. During the outgrowth of different clonal populations, mutations in certain genes (onset mutations) could give rise to multiple genetic events in the same gene (composite mutations). These composite mutations can occur between independent clonal populations, as shown in this figure, or can occur within individual cells. (B) Pie chart showing the distribution of 9636 primary tumor samples over 16 different tumor types, collected from The Cancer Genomic Atlas (TCGA). (C) Lollipop figures show two examples of composite mutations with their corresponding TCGA number. (D) Flow diagram showing the workflow to identify composite mutations in 9636 samples based on mutation and copy number alteration data. We selected all driver genes in the dataset, and from these genes, we defined the tumor‐driving mutations. Finally, if a sample met the requirements for the tumor‐driving events of one gene, we identified it as a composite case. (E) From the 9636 samples, we could identify in total 458 patients (~ 5%) over 16 tumor types (shown horizontally) consisting of composite mutations in 17 different genes (shown vertically). *PIK3CA* has the majority of composite mutations and is present in almost all tumor types. All other composite genes are specific to certain tumor types.

An interesting kind of restricted evolution occurs when independent driver mutations occur in the same gene (Fig. 1A), which we call composite mutations [11, 12, 26]. Why these mutations occur is currently unknown although selective pressures such as oncogene addiction [27] or stochastic accumulation of tumor driving mutations [28] are obvious mechanisms. A better understanding in the underlying biology might provide more insight why therapy resistance occurs only in certain tumors. Previous work has shown that composite mutations are commonly found in tumors [26, 29, 30]. In our approach, we analyzed the genetic information of tumors from ~ 10 000 patients and found that composite mutations occur in around 5% of patients. Our findings show that composite mutations are commonly found alongside specific co‐mutations, commonly located on the same chromosome. With this information, we could generate a prediction model for composite mutations which could provide therapeutic benefits to subsets of patients.

## 2 Materials and methods

### 2.1 Data

Whole‐exome sequencing and copy number variation (CNV) data for each study were obtained from cBioPortal for Cancer Genomics [31] for 16 different tumor types (Fig. 1B); only oncogenes [32] and tumor suppressor genes [33] were selected for the analysis. Given that different tumor types have different mutational frequencies, we normalized the mutation rate by setting a threshold for co‐mutations at 1.04 times the background mutation rate, that is, where the slope showed was most flattened. Hence, this defined co‐mutations that were found together with composite mutations. We focused only on the cancer hotspot mutations [18, 34, 35] and CNVs with a deep deletion or high‐level amplification. Composite mutations are defined by having two independent mutations/CNVs in the same gene in case of an oncogene or three independent mutations/CNVs in the same gene in case of a tumor suppressor gene; co‐mutations are defined as single mutations or CNVs of genes in tumors with composite mutated genes; background mutations are defined as *bona fide* mutations above the driver mutation level. To visualize the distribution of composite mutations within genes, we used the lollipops software [36]. For all the composite mutations, we extracted the nucleic acid changes (A>C, A>T, C>G, A>G, C>A, C>T) from cBioPortal. The distribution per tumor type was visualized after normalizing the frequency to the total number of nucleic acid changes per tumor.

### 2.2 Co‐mutation analysis

#### 2.2.1 Statistical analysis

From all the samples containing a composite mutation, we selected all other mutations in the same sample (defined as co‐mutations, see Section 2.1). To investigate the relationship between the co‐mutations and the composite mutations, we first split the data into two groups: samples containing a specific composite mutation and samples with no composite mutation (non‐composite cases). For each co‐mutated gene, we performed statistical analysis (Fisher's exact test) between these two groups and selected only the co‐mutated genes with a *P*‐value < 0.05. This analysis was done per tumor type and for each composite gene separately. All statistical analyses were performed with r version 3.0.1.

#### 2.2.2 Gene ontology

For the gene ontology analysis, we generated the generic enrichment map (GEM) for all the significant co‐mutated genes per composite gene using the gprofiler software [37].

#### 2.2.3 Chromosomal location disruption

The locations of all the co‐mutated genes and genes with composite mutations were extracted from the Ensemble GRCh38 database. We calculated per gene with a composite mutation the frequency of all co‐mutated genes and normalized it to the gene with a composite mutation. For the randomization analysis, we randomly defined *X* number of new genes with composite mutations.

### 2.3 Prediction model

#### 2.3.1 Input data

We selected the top four interesting genes with composite mutation based on high frequency and relevance toward targeted therapy (*BRAF*, *EGFR*, *KRAS*, and *PIK3CA*). We divided the data into tumor types that have composite mutations in either *BRAF*, *EGFR*, *KRAS*, or *PIK3CA*. For all these separated groups, we defined all the cases that contain composite mutations or without any composite mutations. Per group of genes with a composite mutation, only the significant co‐mutations are taken along as features. These features contain either information about mutation or copy number variation (binary input) or the distance from co‐mutation to the gene with composite mutation (continuous value). All the relative distances of the input belonging to the chromosomal distance model are normalized toward the total distance of the genome (3 000 000 000). See Table S1A for all the input data.

#### 2.3.2 Random forest machine learning

The random forest machine learning algorithm was chosen as it is robust and capable of processing high‐dimensional datasets. Furthermore, it applies to binary data as well as data containing continuous values. This machine learning algorithm builds many decision trees, using bagging and feature randomness to create each tree. By combining all these decision trees (an uncorrelated forest of trees), the most accurate prediction is expected in comparison with a single tree. The library randomforestsrc [38] in R is used to build the random forest model with 100 trees. The training set (2/3) and test set (1/3) are created by dividing the data using createdatapartition from the library caret [39]. All the default parameters are used for building the model.

#### 2.3.3 Ensemble learning

We built separate models for the data for the complementary relationship and the chromosomal disruption. The method of ensemble learning allows us to combine different models to find a more powerful prediction result. We computed a weighted‐average prediction ensemble model. The probability scores given by the separate random forest models were extracted. As there was a stronger complementary relationship, we multiplied the probability score of this model by 0.75, whereas the chromosomal disruption model was multiplied by 0.25. The highest average score defined the final predictive label.

#### 2.3.4 Performance

First, the false‐negative (FN), false‐positive (FP), true‐negative (TN), and true‐positive values were defined by comparing the predicted label and the actual label. From there, the true‐positive rate (TPR), false‐positive rate (FPR), sensitivity, specificity, accuracy, and balanced accuracy were calculated (see formula below). The TPR and FPR were plotted to create the receiver operator curve (ROC) as a performance representation of the prediction model. From the ROC, we calculated the area under the curve (AUC) values (see formula below). The confusion matrixes contain the TP, TN, FP, and FN.

$$\text{Accuracy}=\frac{\mathrm{TN}+\mathrm{TP}}{\mathrm{TN}+\mathrm{TP}+\mathrm{FP}+\mathrm{FN}},$$

$$\text{Balanced accuracy}=\frac{\frac{\mathrm{TP}}{\mathrm{TP}+\mathrm{FN}}+\frac{\mathrm{TN}}{\mathrm{TN}+\mathrm{FP}}}{2},$$

$$\text{Precision}=\frac{\mathrm{TP}}{\mathrm{TP}+\mathrm{FP}},$$

$$\mathrm{TPR},\text{recall},\text{sensitivity}=\frac{\mathrm{TP}}{\mathrm{TP}+\mathrm{FN}},$$

$$\mathrm{FPR}=\frac{\mathrm{FP}}{\mathrm{TN}+\mathrm{FP}}.$$

#### 2.3.5 Randomized data

To evaluate our prediction model, we randomly shuffled the features of each dataset from 10% to 100% in 10 steps. From the shuffled data, we also built a separate random forest model and got the final prediction for each dataset that was received after the ensemble learning. For each shuffled data, the performance was calculated and plotted.

### 2.4 Tumor microenvironment analysis using gene signatures

To see whether composite mutations are correlated to processes in the tumor microenvironment, we analyzed whether gene signatures as published by Thorsson et al. [40] are enriched using a double‐sided *t*‐test for either composite mutated genes using genes (*BRAF*; *n* = 42; *CDH1*; *n* = 2; *CDKN2A*; *n* = 2; *CTNNB1*; *n* = 5; *EEF1A1*; *n* = 4; *EGFR*; *n* = 60; *ERBB2*; *n* = 11; *ERBB3*; *n* = 2; *FGFR3*; *n* = 3; *HRAS*; *n* = 8; *KIT*; *n* = 3; *KRAS*; *n* = 35; *MTOR*; *n* = 3; *NRAS*; *n* = 10; *PDGFRA*; *n* = 2; *PIK3CA*; *n* = 160; *PPP2R1A*; *n* = 2; *PTEN*; *n* = 5; *RRAS2*; *n* = 2; *SMAD4*; *n* = 6; *TP53*; *n* = 28; *VHL*; *n* = 12; compared to *n* = 7216 non‐composite mutated controls) or tumor types in which composite mutations have occurred (prostate; *n* = 2; skin; *n* = 68; lung; *n* = 74; breast; *n* = 70; liver; *n* = 8; uterus; *n* = 37; head and neck; *n* = 31; brain; *n* = 52; bowel; *n* = 12; bladder; *n* = 5; esophagus; *n* = 28; pancreas; *n* = 11; kidney; *n* = 16; cervix; *n* = 11 versus *n* = 7208 tumors without composite mutations). Multiple testing correction was performed by Bonferroni correction, compensating for the number of potential false‐positive signatures (*n* = 64).

### 2.5 Human tissues and ethics statement

The analysis of paraffin materials from patients (*n* = 54) by panel sequencing was covered under the approval of the institutional Biobank review board at the Amsterdam UMC location VUMC. The Biobank license number for approval of the studies is BUP2020‐68 Gene atlas. The study methodologies conformed to the standards set by the Declaration of Helsinki. Patient materials were obtained after routine diagnostics, coded according to the National Code for the Good Use of Patient Material, and were exempt from informed consent because obtaining individual consent would be impractical or even impossible. All identity information is carefully removed or encrypted, and the risk of potential harm or breach of privacy is minimal. The pre‐existing, de‐identified data analysis was performed on samples that had been collected between January 2005 and July 2022.

## 3 Results

### 3.1 Pan‐cancer analysis to identify composite mutations in the cBioPortal for Cancer Genomics database

The discovery of the molecular evolution paths in cancer might benefit the treatment although aggregated genomic data makes it challenging to discover the evolution of the tumor. In this study, we used the publicly available Cancer Genomics database cBioPortal to find common patterns between tumor types. We focused on composite mutations, that is, independent driver mutations that have occurred at least twice in the same gene, which can be considered as a footprint of independent genetic events and which can be extracted from aggregated data (Fig. 1A).

Molecular evolutionary paths have not been systematically studied on a pan‐cancer scale. We therefore used the following strategy to deconvolute whole‐exome and copy number variation (CNV) data of 9636 patients in 16 tumor types [31] (Fig. 1B, Fig. S1a,b). First, we reduced the number of genes by selecting only the oncogenes and tumor suppressor genes, followed by a selected list of oncogenic driver mutations [18, 34, 35] and deep deletions or high‐level amplifications. We define composite mutations as the presence of at least two oncogenic driver mutations or three inactivating tumor suppressor mutations. As an example, sample *TCGA‐BF‐A3DL‐01* contains the mutations V600E and V600M (both oncogenic driver mutations [18, 34, 35]) in the *BRAF* gene and is thus defined as a case with a composite mutated gene (Fig. 1C, left). Another example is a case with composite mutations in sample *LUAD‐NYU1142‐Tumor*, which has the oncogenic driver mutations T790M and L858R in the *EGFR* gene (Fig. 1C, right). All other mutations or CNVs for each tumor are defined as co‐mutations. This analysis was followed by a frequency and statistical analysis to select the significant co‐mutations (Fig. 1D). In the heatmap of Fig. 1E, we show the 17 genes that contain composite mutations representing 458 patients (~ 5% of all analyzed patients) in 16 tumor types [31]. The tumor types thyroid and cervix did not show any composite mutations and showed a homogenous mutation landscape (80% *BRAF* V600E and 83% a mutation in p53, respectively). All individual oncogenic composite mutations are presented in the lollipop graphs of Fig. S1b; individual mutations are listed in Table S1B–D and the excluded thyroid and cervix datasets in Table S1E.

The gene *PIK3CA* has the majority of composite mutations and is present in almost all tumor types. This gene is observed to be one of the most frequently mutated genes in cancer [41]. Furthermore, composite mutated genes are found to be oncogenes rather than tumor suppressors which is not surprising since copy number losses of both alleles make it impossible to find additional composite mutations in many cases. Mutation signature analysis on composite mutations most commonly showed C>T substitutions, indicative of replication errors and/or mutagen‐induced mutations (Fig. S1c, left panel, red colors).

### 3.2 Validation of composite mutations in cell line data of the Cancer Cell Line Encyclopedia

**Fig. 2:**
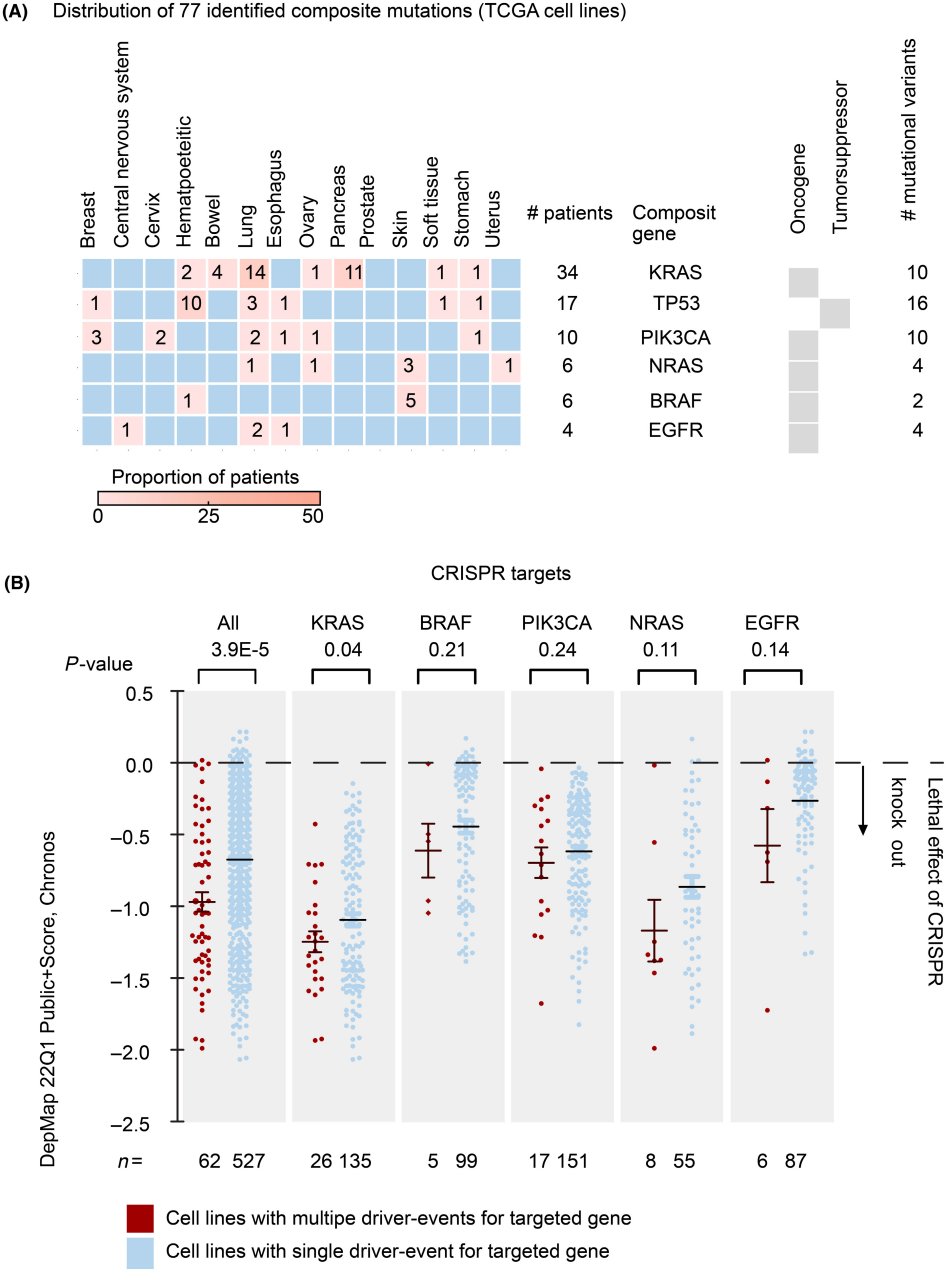
Validation of composite mutations in cell line data also resulted in cell lines with composite mutations. (A) From 1020 different tumor cell lines, we identified a total of 77 cell lines with composite mutations in a total of six genes. *KRAS* has the majority of composite mutations and is present in almost all tumor types. All other composite mutated genes are specific to certain tumor types. (B) Scatterplots showing DepMap (version 22Q1) lethal effects of CRISPR/CAS9 knock‐out experiments, where targeting genes with composite mutations/CNVs showed a higher dependency (*P*‐value = 3.9e‐5) compared to knock‐out of the same genes without composite genetic events. Analysis of individual genes showed the same trend and was significant in the case of KRAS. Error bars indicate the standard deviation. *P*‐values indicate the significance of the difference as calculated with a one‐sided *t*‐test.

To validate our previous findings, we performed the same analysis on the CCLE database on *n* = 1020 cell lines derived from 14 different tumor tissues. Here, we identified in total 77 cases with composite mutations (~ 7% of the cell lines), that is, in the same range as the tumor tissues. Also, frequently composite mutated genes as found in tissues were identified (Fig. 2A), again identifying *PIK3CA* as a frequently affected gene, although we found *KRAS* as the most frequently affected gene, mainly observed in lung and pancreatic tumors. We observed a similar mutation signature, pointing to replication error or mutagen‐induced mutations as the major source of composite mutations (Fig. S2). These data indicate that the patterns of composite mutations that we saw in clinical specimens are similar to what we see in cell lines.

The previous results indicate that cell lines might be a good model system to analyze the consequences of composite mutations. To compare the dependency of cell lines with composite mutations versus cell lines with single mutations in the same genes, we analyzed the DepMap CRISPR screen data as performed on the CCLE cell lines (Fig. 2B). When all driver genes (*KRAS*, *BRAF*, *PIK3CA*, NRAS, and *EGFR*) are grouped, we see that cell lines with composite mutations show a significant viability loss upon their CRISPR/CAS9‐mediated deletion when compared to matched cell lines with single mutations for each selected gene (*P*‐value = 3.9 × 10^−5^). However, when driver genes are analyzed separately, we did not observe significant differences although all data show a trend in the same direction. Both cell lines with composite mutations and cell lines with matched non‐composite mutations show a viability loss, showing that they are oncogenic drivers.

So far, we confirmed the presence of composite mutations found in a substantial fraction of patients and cell lines (5–7%) and that these could have been positively selected based on the viability benefit as determined based on preliminary CRISPR/CAS9 screen data analysis.

### 3.3 Co‐mutations occurring with composite mutations overlap with common mutation profiles seen in cancer

**Fig. 3:**
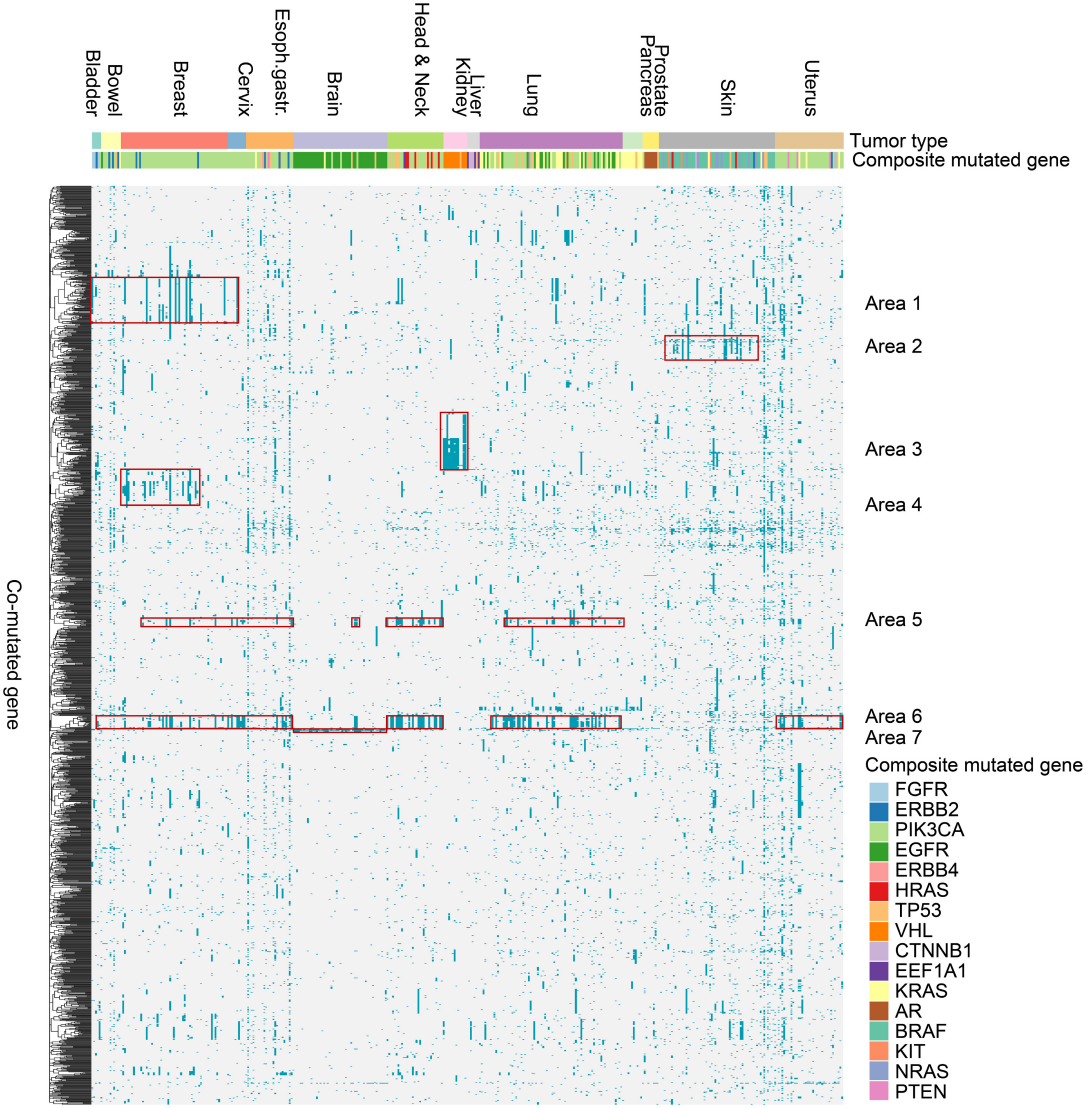
Composite mutations in the same gene correlate with common patterns of co‐mutations. Heatmap showing common patterns of mutations that were found together with composite genetic effects for all patients from the TCGA cohort. Columns represents a patient with a composite mutation/CNV, sorted according to tumor type. Rows represent an unsupervised hierarchal clustering of relevant co‐mutations, revealing a consistency in regions marked in red (i.e., areas of commonly mutated genes as seen in certain tumor types and/or composite genes).

To evaluate whether composite mutated genes might arise within a certain genetic context, we performed a hierarchical cluster analysis of genes co‐mutated with composite mutations. The result of this unsupervised clustering is shown on the vertical axis in Fig. 3. In this clustering, we see that genes co‐mutated with composite mutations are spread over several tumor types, which is expected for commonly mutated genes such as TP53. Besides this diffuse pattern, there are clusters of co‐mutated genes that are linked to either the tumor type and/or the composite mutated gene. These sub‐clusters are indicated by areas on the cluster grid. Gene ontology analysis showed a correlation to genes involved in cell cycle‐related processes (Fig. S3). Most areas of co‐mutated genes are tumor‐specific (areas 2, 3, 4, and 7). In other cases, clusters of genes are seen for different tumor types (areas 1, 5, and 6). Area 5 is interestingly involved in endocrine processes and is present in the tissues belonging to the breast, cervix, esophagus, brain, head and neck, and lung.

These data show that there are common co‐mutations with composite mutations that either are linked to a certain tissue type or linked to the particular composite mutations. This indicates a certain level of conservation of co‐mutations that might set the stage for composite mutations to occur.

### 3.4 Genes showing composite mutations are accompanied by certain co‐mutations that also co‐occur in a non‐composite setting

**Fig. 4:**
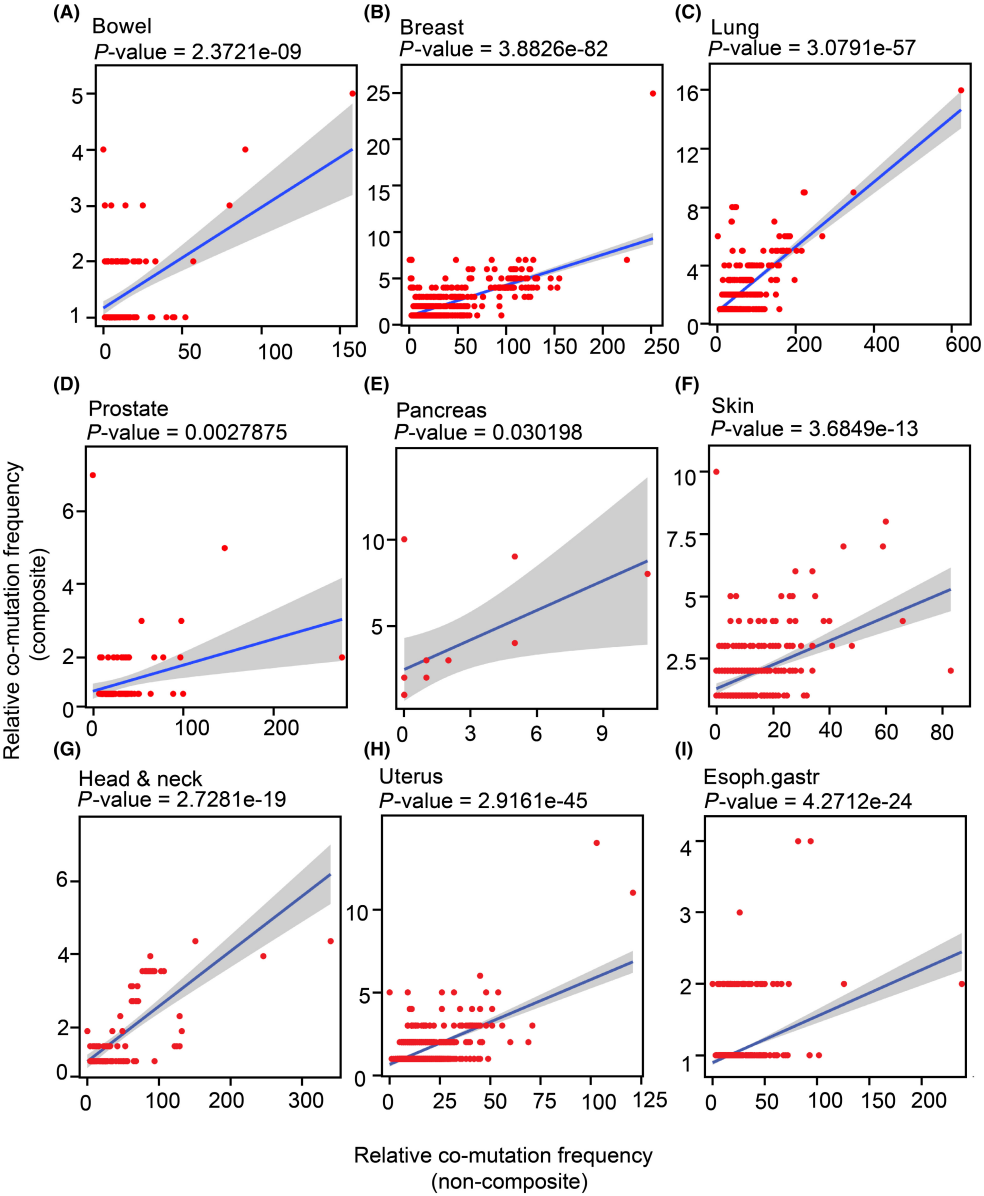
The heterogeneity in co‐mutation profiles shows a complementary relationship. (A–I) Scatterplots showing the correlation between the frequencies of co‐mutations seen in cases with composite mutations (vertical axis) versus cases without composite mutations (horizontal axis). The *P*‐value is based on the direction of the variables of *x* and *y*. A significant correlation between the frequency of pairs of composite mutations with co‐mutations versus the same pairs of mutations in a non‐composite setting is seen. *P*‐values indicate the level of significance of the Pearson correlation.

To follow up onto the previous observation, we assessed whether co‐mutations that commonly occur with composite mutations also co‐occur in a non‐composite setting. For this, the mutation frequency of co‐mutations to composite mutations was compared to the matching mutation frequencies in patients without composite mutations. Given that most co‐mutations are seen in a specific tissue‐type context, we did this analysis within tissue types. In Fig. 4, the tumor types with a significant relationship are shown (*n* = 9, indicated by A–I), where on the horizontal axis the co‐mutation frequencies are presented and on the vertical axis the composite mutation frequency. We observe that the co‐mutation frequency of composite mutations correlates to co‐mutation frequencies in a non‐composite setting. This suggests that composite mutations could arise due to predispositions from the co‐mutation context. Non‐significant relationships or relationships with a slope around zero (*n* = 5) are shown in Fig. S4.

### 3.5 Genetic aberrations are enriched within chromosomal territories of genes with composite mutations

**Fig. 5:**
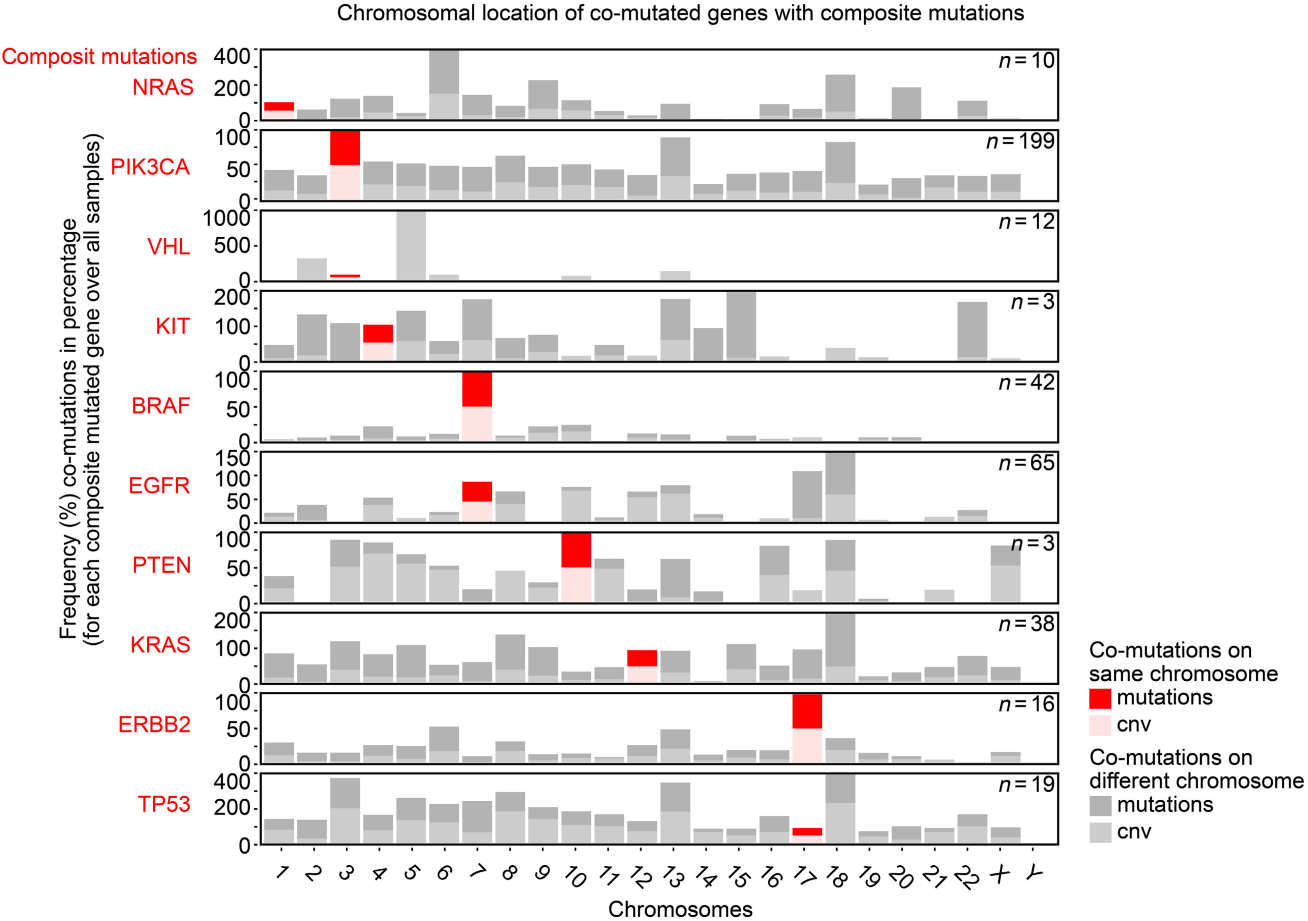
Co‐mutations are commonly localized on the same chromosome as composite mutations. Histograms showing the amounts of all mutations/CNVs on each chromosome for tumors with composite genetic events only. The amount of co‐mutations or CNVs on chromosomes where the composite mutations are located are shown in red, and CNVs are shown in pink. As a reference, mutations are shown for the remaining chromosomes in gray. These results suggest chromosomal areas could be selectively disrupted (chromosysmos), showing increased local mutation rates resulting in multiple mutations in the same gene, particularly for the genes *PIK3CA*, *BRAF*, *PTEN*, and *ERBB2*.

In addition to the observation that tumors with composite mutations often show the same co‐mutated genes, we also observed that co‐mutated genes are commonly in close chromosomal vicinity with composite mutations (Fig. 5). To rule out artifacts in this interpretation, we performed a randomized analysis where we took other, non‐composite, tumor‐driving genes and performed a similar analysis. This analysis did not show a similar relationship (Fig. S5A). We mapped the chromosomal locations of co‐mutations to the composite mutated genes which showed that in many cases, the co‐mutations occur on the same chromosome that shows composite mutations. Indeed, 35% of co‐mutations that occur in the presence of composite mutations are localized on the same chromosome over an average expectancy of 4.2% per chromosome for non‐composite mutations (*P* < 0.00001). We analyzed whether the relation was found due to large areas of chromosomal gains and losses, which is not likely since mutations and CNVs occur rather in a mutually exclusive way in tumors with composite mutations (Fig. S5B). Furthermore, the chromosomal enrichment of mutated or CNV affected genes with composite mutations is significant compared to the background, with a *P*‐value of 0.02 and 1.9 × 10^−5^, respectively (Fig. S5C). This suggests that the chromosomal area around the composite mutation is predisposed to gain mutations and/or CNVs, making it more likely to obtain a secondary mutation in the same gene. We propose to call this phenomenon *chromosysmos* (i.e., earthquake areas of the genome), a phenomenon not described before to our knowledge.

### 3.6 Composite mutations can be predicted based on co‐mutations and chromosomal location

**Fig. 6:**
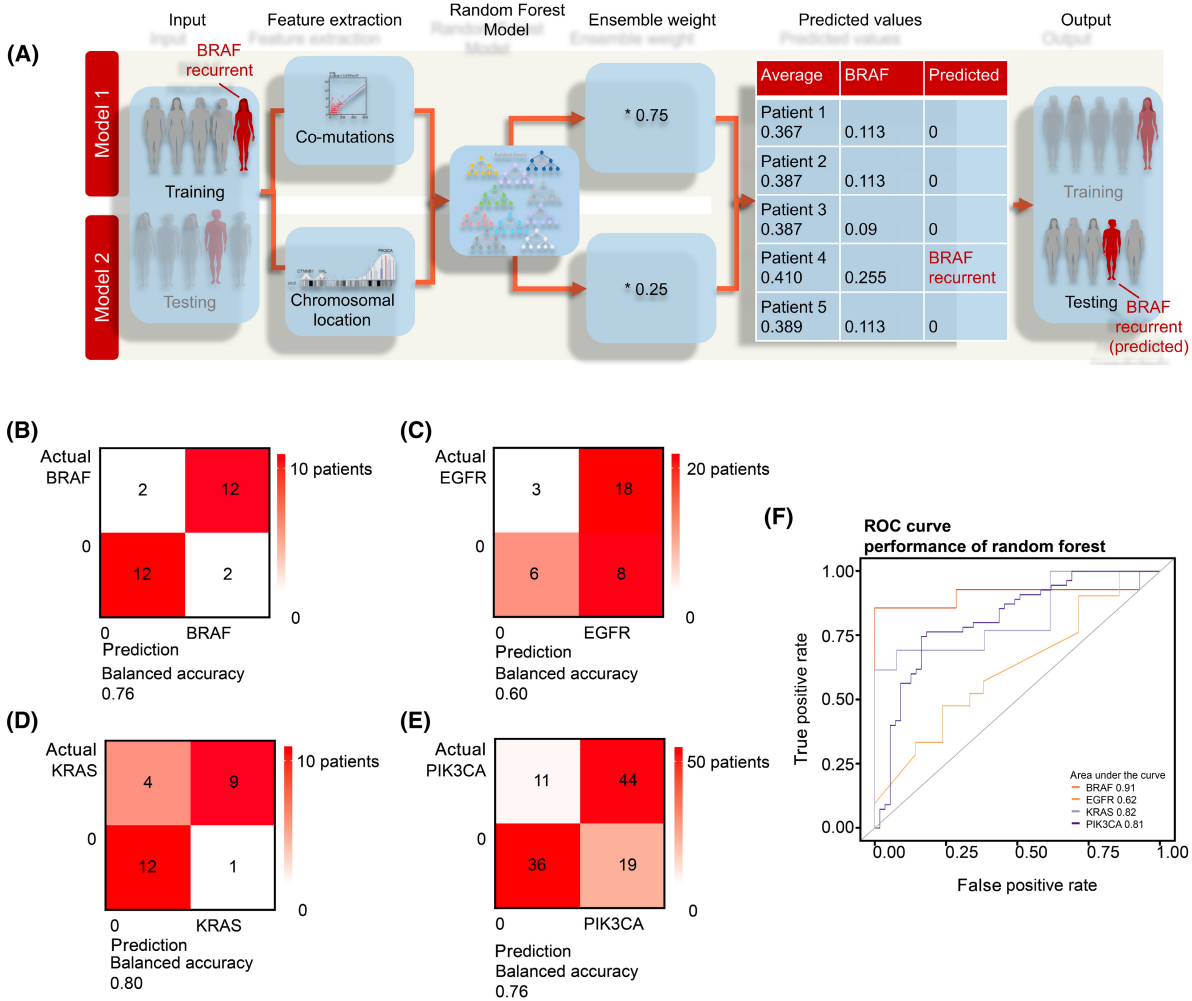
Random forest model to predict composite mutations. (A) Machine learning workflow showing that patient DNA mutation profiles that contain composite mutations/CNVs in *BRAF*, *EGFR*, *KRAS*, and *PIK3CA* were matched to patterns of co‐mutations/CNVs (binary input, based on mutations) and chromosomal location (continuous value, based on the distance of co‐mutation toward a composite mutated gene) and divided per gene (*BRAF*, *EGFR*, *KRAS*, or *PIK3CA*). Both types of data were provided to a random forest model, and the probability measures were used in ensemble learning. In ensemble learning, we put a weight of 0.75 on the result of the co‐mutation frequency, as these features were more important than the chromosomal mutational distance (weight of 0.25). From the maximum value of the average of both weighted probabilities, the final predictive label was defined. (B–E) Confusion matrices showing the performance of the random forest model for *BRAF*, *EGFR*, *KRAS*, and *PIK3CA*, respectively, providing an overview of the TP, TN, FP, and FN values. Besides *EGFR*, all the other models resulted in a well‐balanced accuracy. (F) ROC curves showing the performance for all the models.

Composite mutations might be present in subpopulations of tumor cells but not necessarily detected in a biopsy sample. Therefore, the prediction of the occurrence of composite mutations might have translational value since these could drive therapy resistance. Using the previous observations, we generated a prediction model for composite mutations in four genes that most frequently show composite mutations (*BRAF*, *EGFR*, *KRAS*, and *PIK3CA*, Fig. 6A). We collected the DNA mutation profiles of the patients for each gene separately and created the data for the complementary relationship (binary input, based on mutations) and chromosomal disruption (continuous value, based on the distance of co‐mutation toward a composite mutated gene). Both datasets were trained by the random forest, and the probability measures were used in ensemble learning. In ensemble learning, we put a weight of 0.75 on the result of the complementary relationship, as this result was much stronger than the chromosomal disruption (weight of 0.25). From the maximum value of the average of both weighted probabilities, the final predictive label was defined. The performance of the model is presented in a confusion matrix (Fig. 6B–E) and a responder operator curve (ROC) curve (Fig. 6F). The confusion matrix of all genes shows no imbalance in prediction, and there is a similar distribution between composite mutations and no composite mutations. This suggests that the model can predict composite mutations as well as no composite mutations. The ROC curve shows the performance based on TPR and FPR, with an area under the curve of *BRAF* 0.91, *EGFR* 0.62, *KRAS* 0.82, and *PIK3CA* 0.76. Finally, we evaluated the model with randomized data, which resulted in a worse performance when data were randomized by at least 70% (Fig. S6A, individual genes are shown in Fig. S6B–E, ROC figure is shown in Fig. 6F, and see also Table  S2A–C for performance metrics and randomization effects).

To evaluate whether the tumor microenvironment might drive composite mutations, we used gene signatures published by Thorsson et al. [40]. We made comparisons between (a) all composite mutated versus negative tumors, (b) tumors representative of individual composite mutated genes versus negative tumors, and (c) tissues that have composite mutations versus negative tissues. Although several significant differences were found, we have not been able to show that these signatures showed a consistent pattern over the three comparisons (Fig. S7A,B). This indicates that either no global role of the microenvironment in the generation of composite mutations exists or that the power of the data is currently insufficient to find these patterns. When taken together, our results indicate that composite mutations can be predicted from individual mutation patterns without being directed by the microenvironment.

To show the relevance of our work in the clinic, we searched in our local specimens whether we were able to identify composite mutations in patients. We were able to identify 54 patients with two up to four tumor‐driving mutations in the *EGFR* gene (Table S3). This indicates that the methodology could have value in a clinical setting.

## 4 Discussion

Treatment of cancer patients is increasingly guided by mutations in the DNA of their tumors. In the future, it might be possible to make predictions of the prospective molecular evolution and its effect on the therapy response of tumors based on currently obtained DNA information. As a proof of concept, we provide here an approach to predict multiple mutations in the same gene as a marker for mutations that have occurred independently in time. For this, we collected data from the cBioPortal for Cancer Genomics database and found that approximately 5% of all tumors contain composite mutations in the same gene. These composite mutations primarily occur in kinases or other tumor‐driving genes, matching the COSMIC's cancer mutation census. Composite mutations are found in tissues where mutations of the respective genes commonly arise in a non‐composite fashion, for example, *PIK3CA* (breast, lung, and uterus); *EGFR* (lung and brain); *KRAS* (lung and pancreas); *BRAF* and *NRAS* (skin); *VHL* (kidney); and *SMAD4* (prostate). The composite mutations as seen in tumor tissues were independently observed in cell line data of the CLLE database which showed a similar outcome (7% composite mutations), affecting the same genes albeit in different relative frequencies, which might be explained by differences in mutation profiles of tissue and cell lines [42]. By matching cell line data to CRISPR‐Cas9 data, we could show that knock‐out of composite mutated genes has a stronger viability effect than knock‐out of their respective single mutated cases, implicating composite mutations as a mechanism toward enhanced oncogene addiction.

We argued that composite mutations might arise as a result of a predisposition due to the genetic context where they can occur. Indeed, mutations that are commonly seen as combinations in tumors correlate to the occurrence of composite mutations and their co‐mutations in many tissues. These findings match observations by Turajlic et al. for renal cancer [12]. In addition, we saw that many genes that are co‐mutated are in close chromosomal vicinity with composite mutations. We mapped the chromosomal locations of co‐mutations to the genes with composite mutations which showed that in many cases, the co‐mutations occur on the same chromosome that shows composite mutations. Indeed, a third of co‐mutations that occur in the presence of composite mutations are localized on the same chromosome which is highly enriched over a random distribution of mutations. We call this phenomenon *chromosysmos*, that is, “Earthquake” areas in the genome where high levels of mutations are observed.

These data indicate that composite mutations show a strong correlation to common co‐mutations and that co‐mutations commonly occur in the same chromosomal area as the composite mutation. We argued that it should be possible to predict composite mutations based on co‐mutations and their chromosomal location. We generated a random forest model to predict heterogeneous mutations in *BRAF*, *EGFR*, *KRAS*, and *PIK3CA* genes, for which sufficient data were available. All four generated models could predict the presence of a composite mutation. The model for EGFR was considerably weaker because it showed a false‐positive outcome. The robustness of the models was confirmed with a stability test.

A similar approach has been proposed to be linked to the total level of heterogeneity in the tumor based on transcriptomics [43], although our approach is not proposed to be generalizable to tumor heterogeneity *per se* given its strong links to patterns of co‐mutations and chromosomal locations. Rather, we think that our approach could impact several personalized therapies where heterogeneous mutations of tumor‐driving genes can cause therapy resistance. Previous studies have indicated that the microenvironment could direct composite mutations [44]. We have tested this hypothesis based on mRNA expression analysis and did not find clear indications that the composite mutations are correlated to processes in the tumor microenvironment. The power of our analysis might have been insufficient to observe these patterns.

Ideally, we should validate our model on independent data besides the cell line data. However, this turned out to be difficult since there is no similarly sized dataset available, where both CNV and mutation data are integrated. In contrast, other sources, such as the PCAWG and Hartwig Foundation datasets, lack CNV data. Different methods might use other thresholds for mutation calling, which might lead to biases in detecting mutations. Some larger datasets are based on panel sequencing (such as the MSK data) further complicating cross‐validation since not all mutated or amplified genes can be taken into account. The strength of our model is that we analyzed an extended dataset representing different tumor types which show consistent mutation patterns by both frequency and chromosomal location. Moreover, these observations were independently confirmed in cell line data. To improve our prediction model, a follow‐up study could be done, for example, using our in‐house samples.

## 5 Conclusions

Therapy resistance is a major threat to effective cancer treatment, and predictive molecular profiles might assist in selecting patient cohorts that are more likely to benefit from a chosen treatment based on their predicted molecular evolution. Therefore, we think that our approach forms a step to anticipate tumor evolution by predicting conserved genetic paths, which might translate into more consistent outcomes of targeted therapies.

## Conflict of interest

The authors declare no conflict of interest.

## Author contributions

BAW, CLB, and LEAB developed the conceptual framework. AK conducted all bioinformatics analyses. CLB and LEAB performed curation of relevant literature. TR was involved in the collection and analysis of the clinical samples. TW, RV, and DN contributed to the conception and edited the manuscript. BAW supervised the work. AK, CLB, and BAW wrote the paper. All authors reviewed the paper.

### Peer review

The peer review history for this article is available at https://www.webofscience.com/api/gateway/wos/peer‐review/10.1002/1878‐0261.13636.

## Supporting information


**Fig. S1.** Overview of copy number variation and mutation data of the datasets, individual composite mutations and mutation signatures of composite mutations as analyzed.
**Fig. S2.** Mutation signature analysis on the cell line data did not show a clear difference between composite mutations and other mutations.
**Fig. S3.** Composite mutations correlate with common patterns of co‐mutations.
**Fig. S4.** The heterogeneity in co‐mutation profiles shows a complementary relationship.
**Fig. S5.** Validation co‐mutations are enriched within the chromosomal territory of genes with composite mutations (chromosysmos).
**Fig. S6.** Evaluation of Random Forest model to predict composite mutations.
**Fig. S7.** Composite mutations do not seem to be driven by the tumor microenvironment.

**Table S1.** Data overview.

**Table S2.** Performance of the RF model.

**Table S3.** In‐house identification of composite mutations in the EGFR gene.

## Data Availability

All public sources used for the project are provided in Table S1. Scripts are available at: https://github.com/bartwesterman/composite‐mutations.
