# Supplementary material for: Oncogenic composite mutations can be predicted by co‐mutations and their chromosomal location
Source: Mol Oncol. 2024 May 16;18(10):2407–22. doi: 10.1002/1878-0261.13636 (PMC11459034; doi:10.1002/1878-0261.13636)
Supplement: Supplementary file 1 — Fig. S1. Overview of copy number variation and mutation data of the datasets, individual composite mutations and mutation signatures of composite mutations as analyzed. Fig. S2. Mutation signature analysis on the cell line data did not show a clear difference between composite mutations and other mutations. Fig. S3. Composite mutations correlate with common patterns of co‐mutations. Fig. S4. The heterogeneity in co‐mutation profiles shows a complementary relationship. Fig. S5. Validation co‐mutations are enriched within the chromosomal territory of genes with composite mutations (chromosysmos). Fig. S6. Evaluation of Random Forest model to predict composite mutations. Fig. S7. Composite mutations do not seem to be driven by the tumor microenvironment. [file MOL2-18-2407-s004.docx]

##

Contents

[Supplementary Figure 1 2](#_Toc155616504)

[(continued) 3](#_Toc155616505)

[(continued) 4](#_Toc155616506)

[(continued) 5](#_Toc155616507)

[(continued) 6](#_Toc155616508)

[(continued) 7](#_Toc155616509)

[Supplementary Figure 2 9](#_Toc155616510)

[Supplementary Figure 3 10](#_Toc155616511)

[Supplementary Figure 4 11](#_Toc155616512)

[Supplementary Figure 5 12](#_Toc155616513)

[(continued) 13](#_Toc155616514)

[Supplementary Figure 6 14](#_Toc155616515)

[Supplementary Figure 7 16](#_Toc155616516)

## Supplementary Figure 1


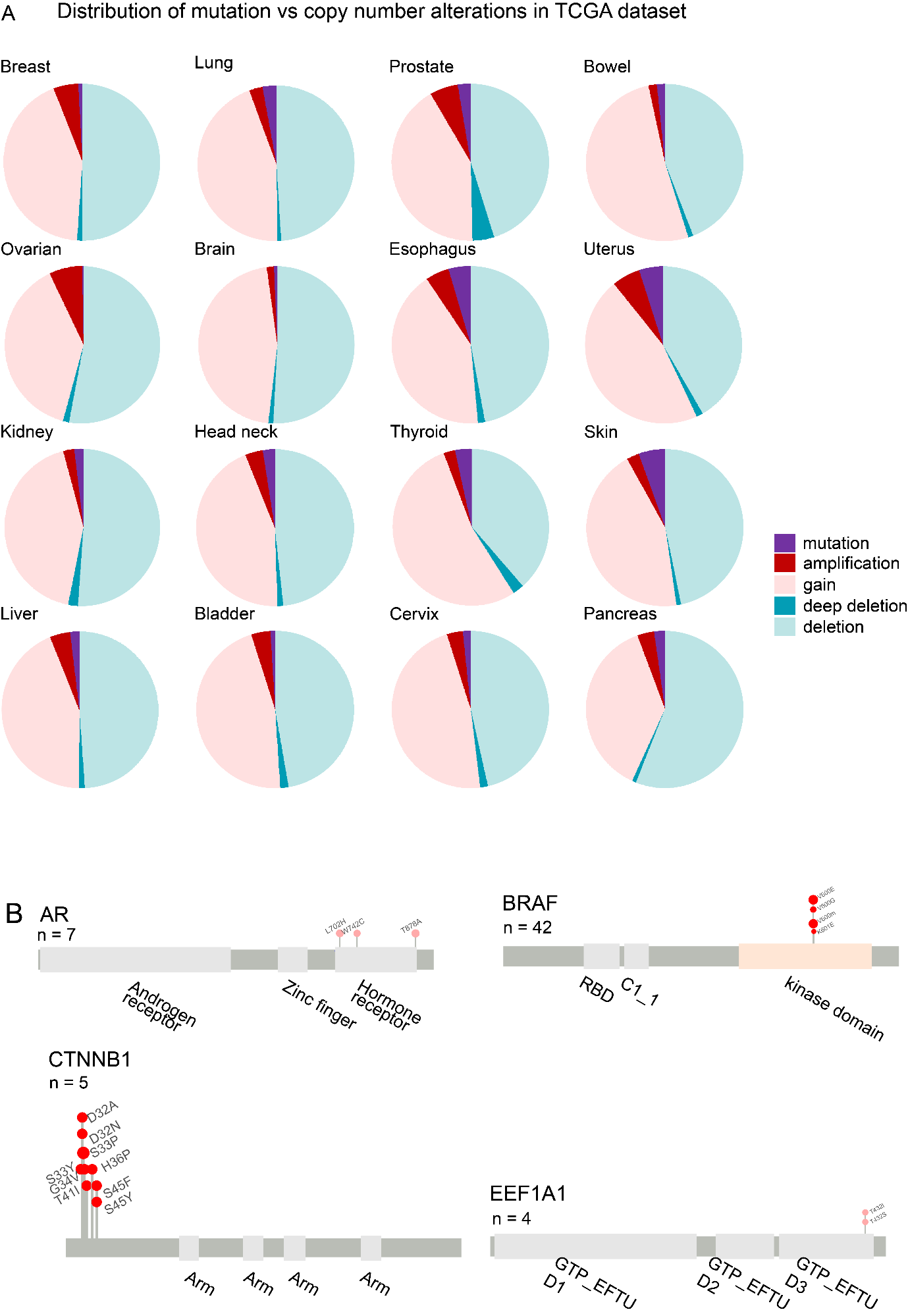


### (continued)


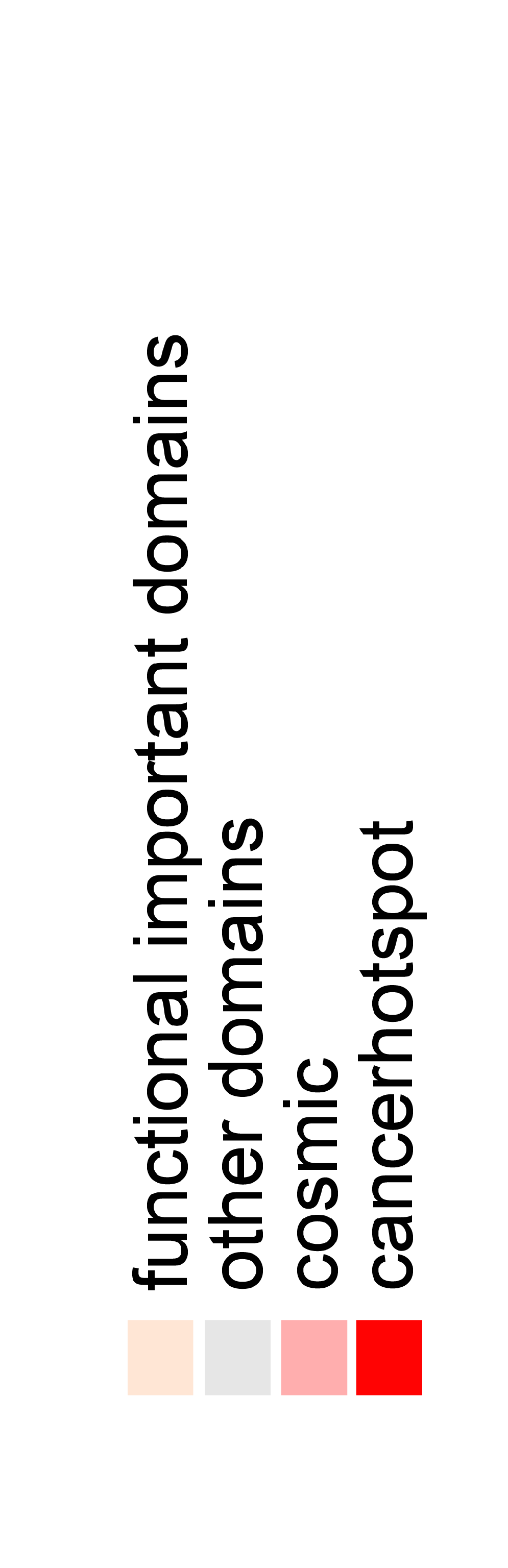

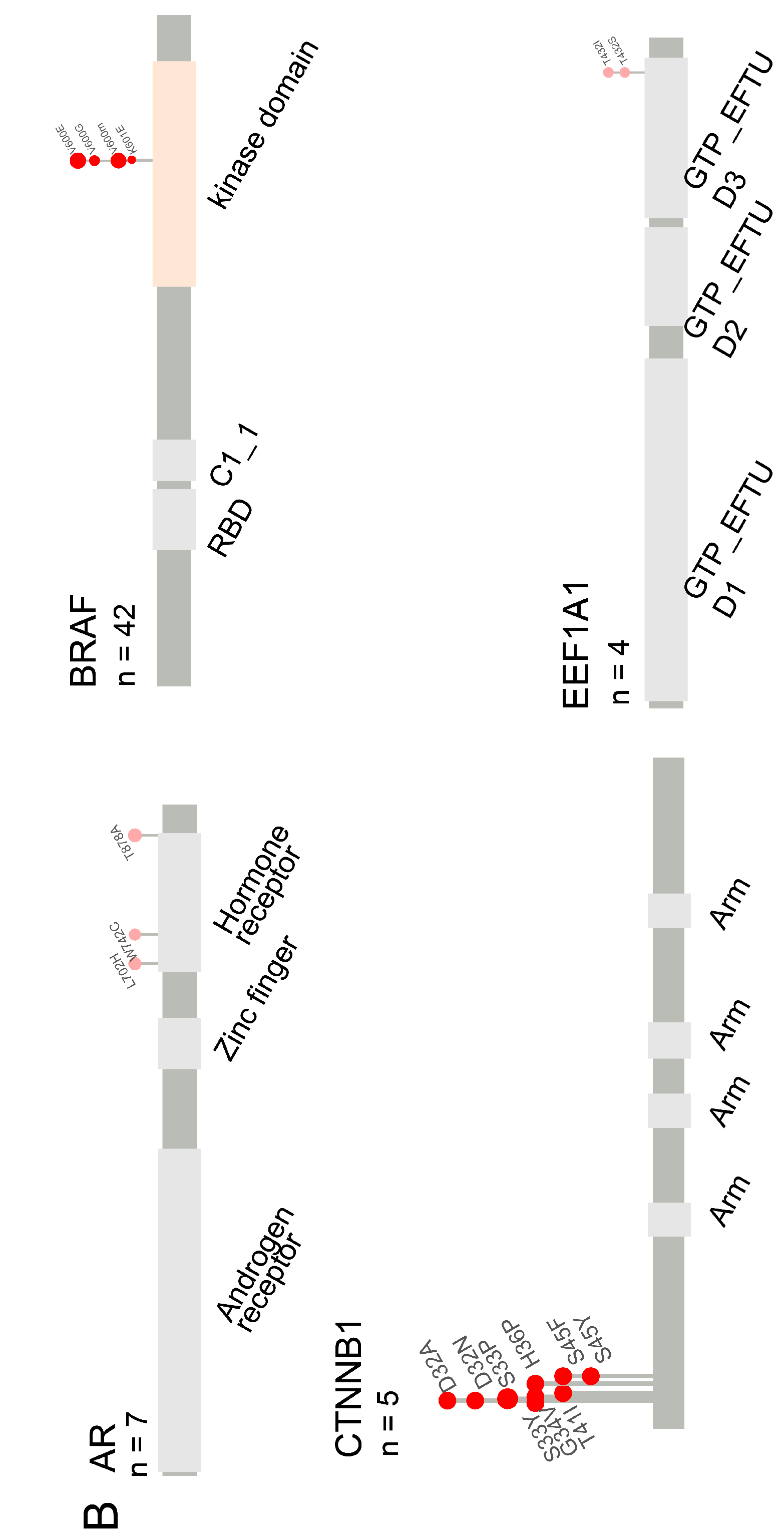


### (continued)


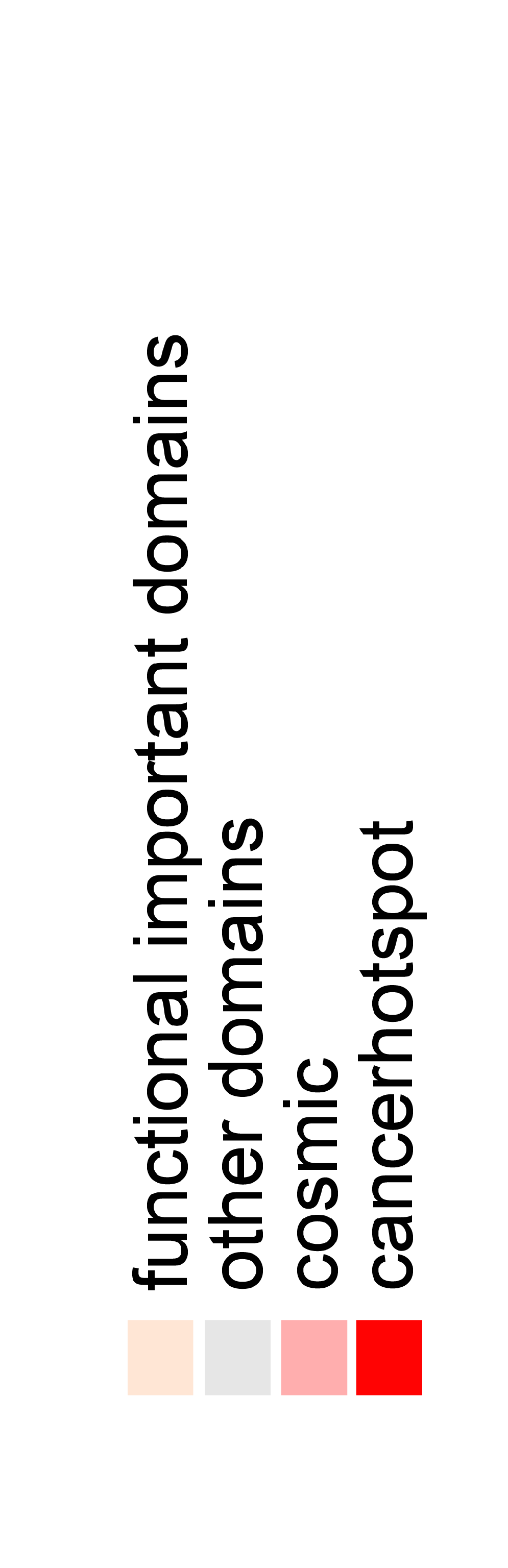

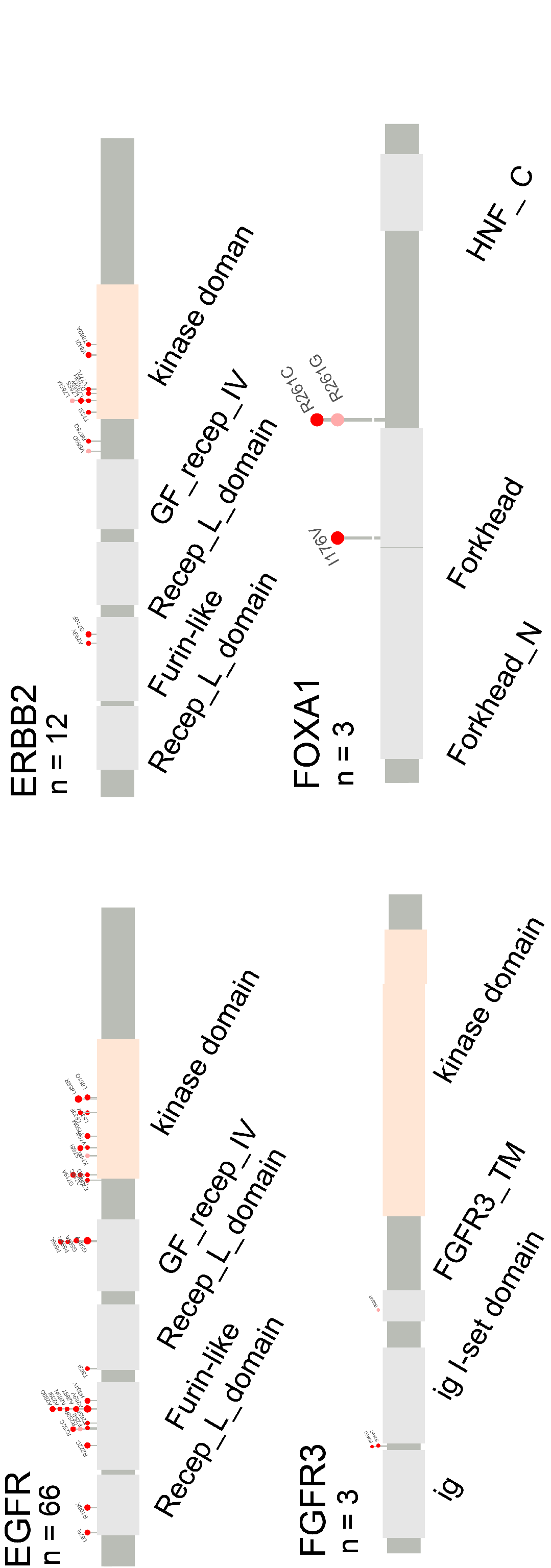


### (continued)


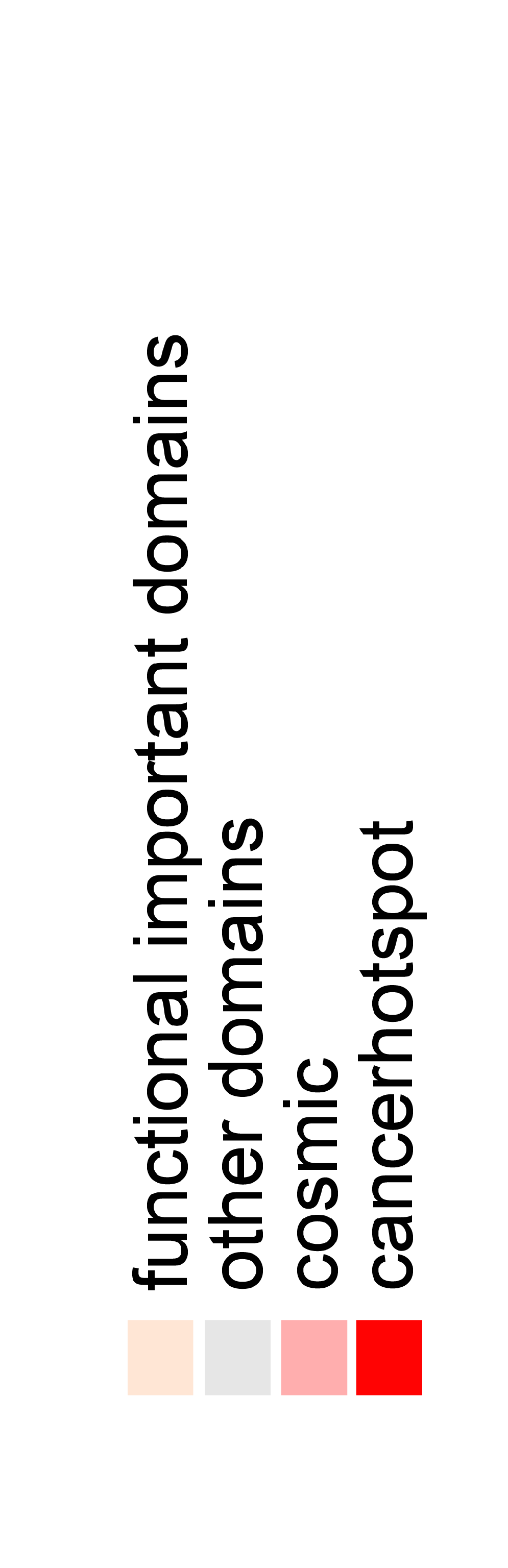

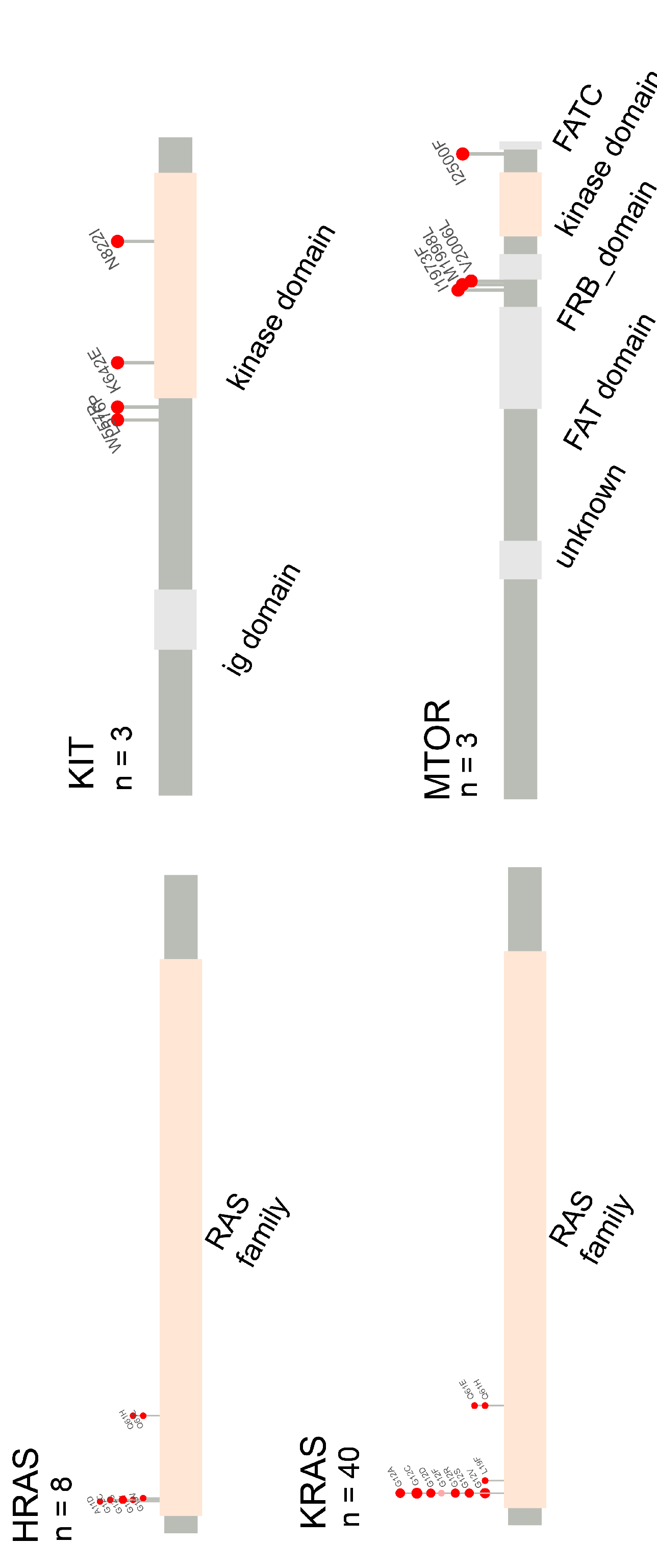


### (continued)


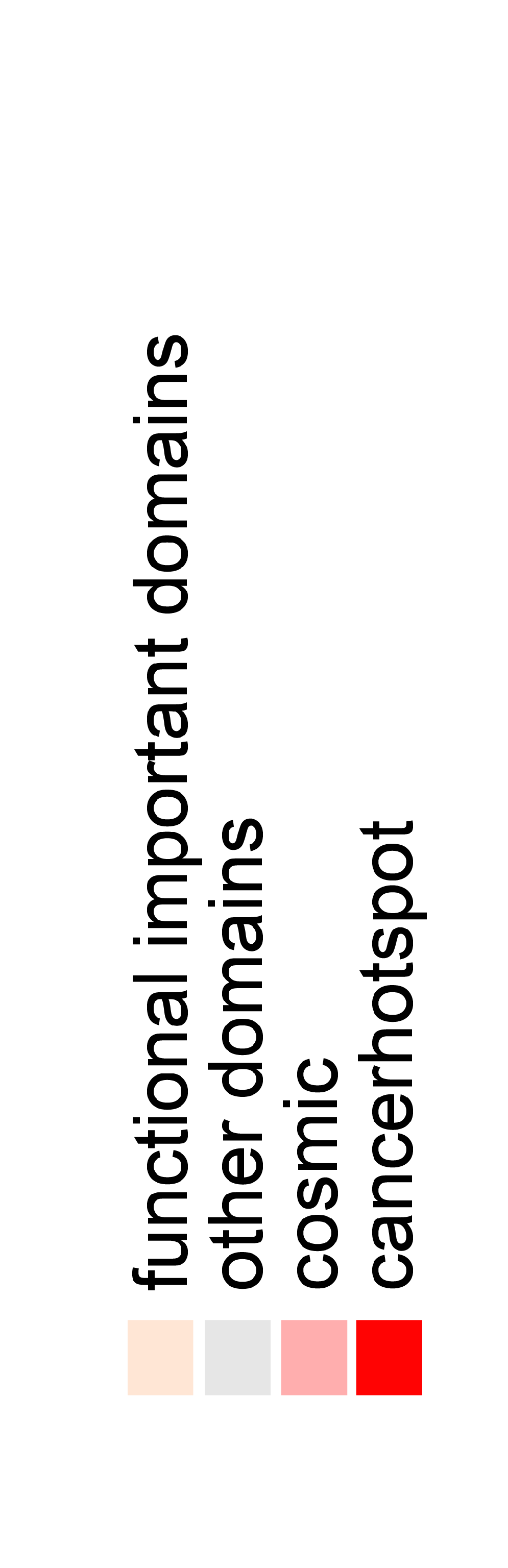

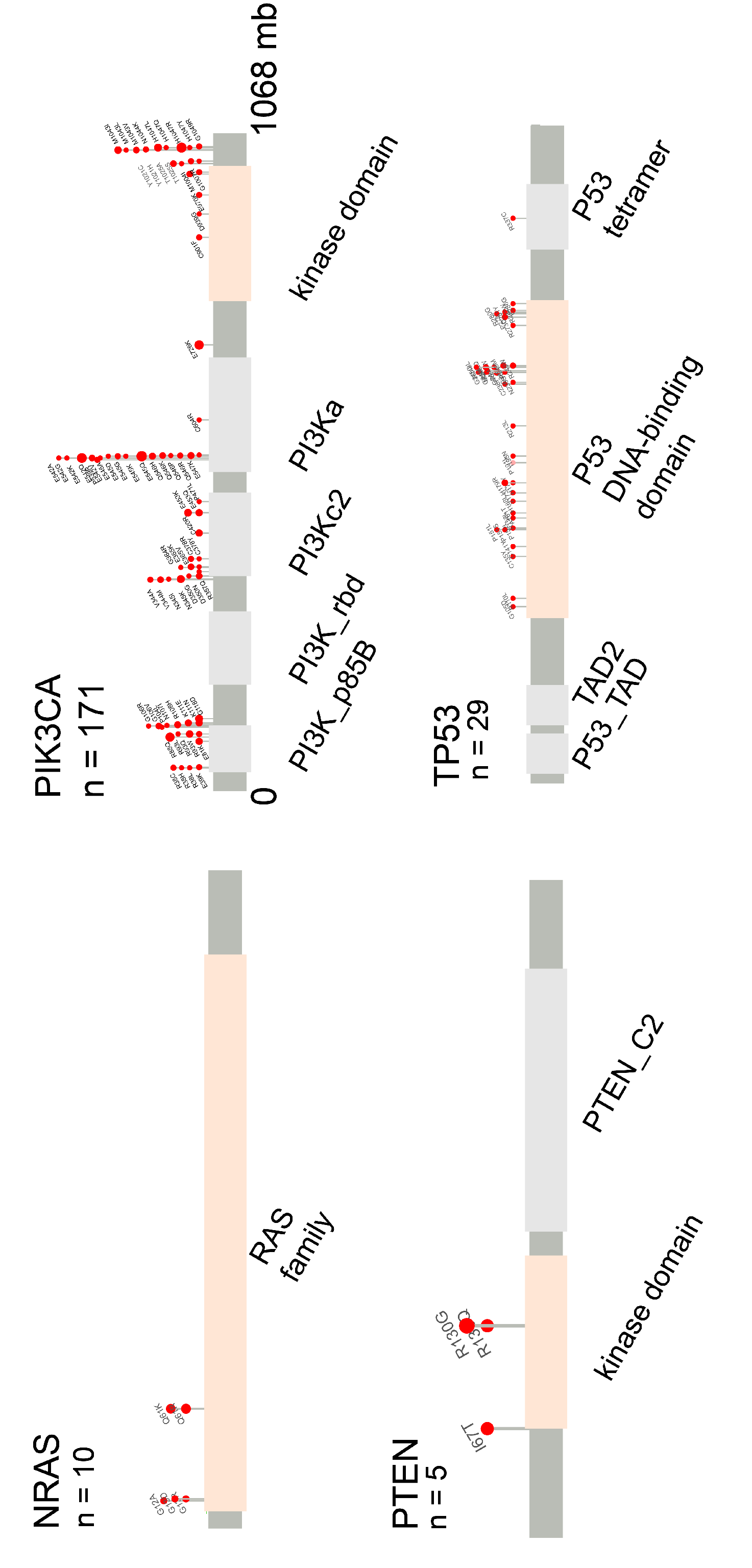


### (continued)


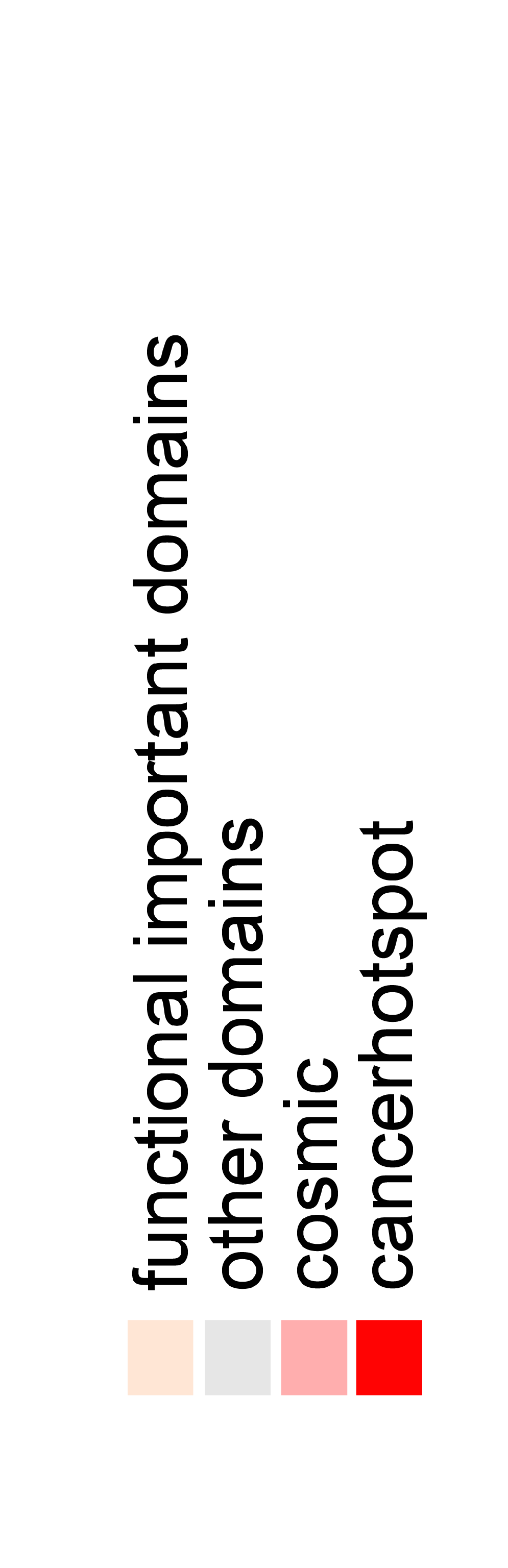

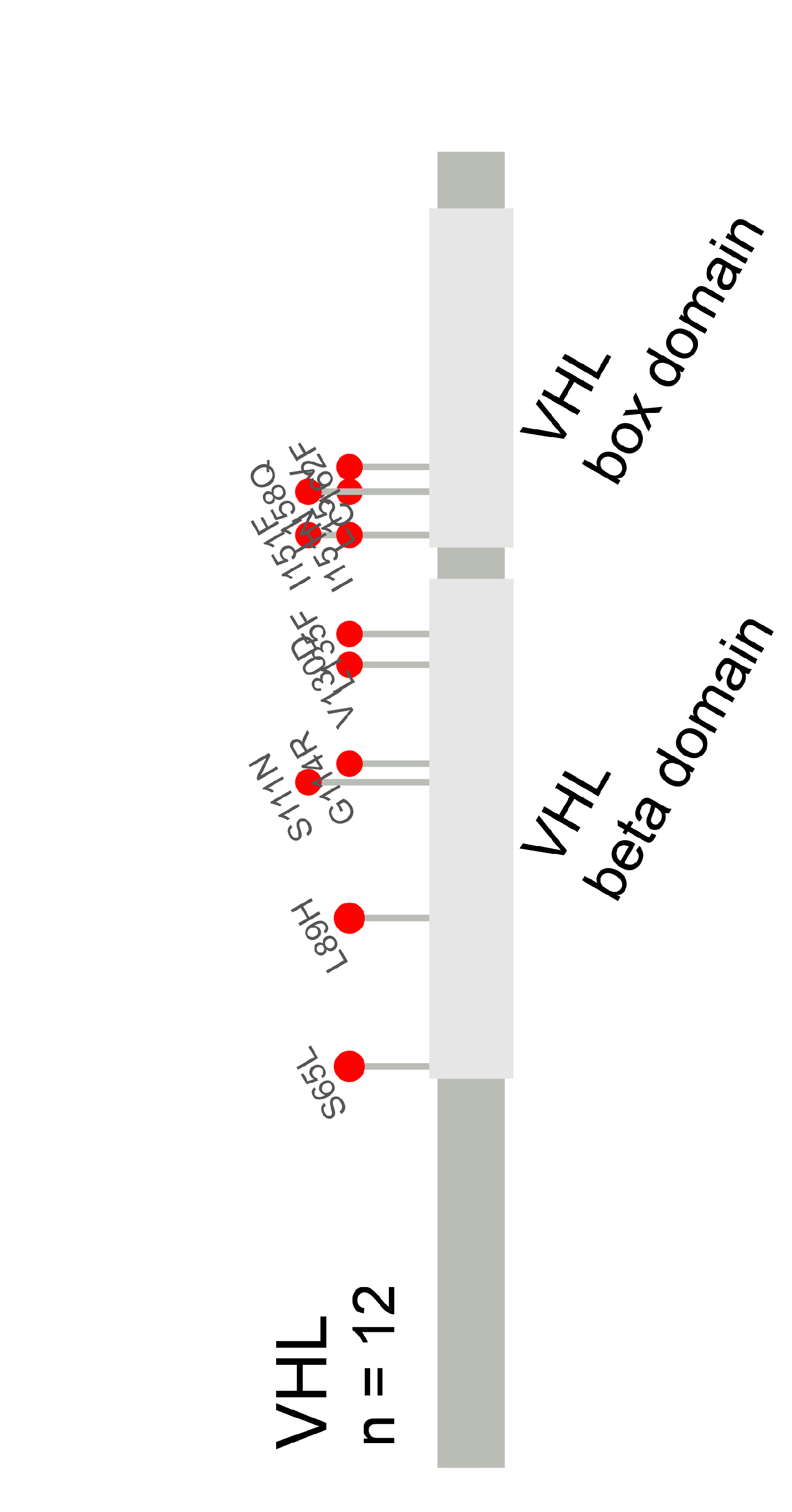
(continued)


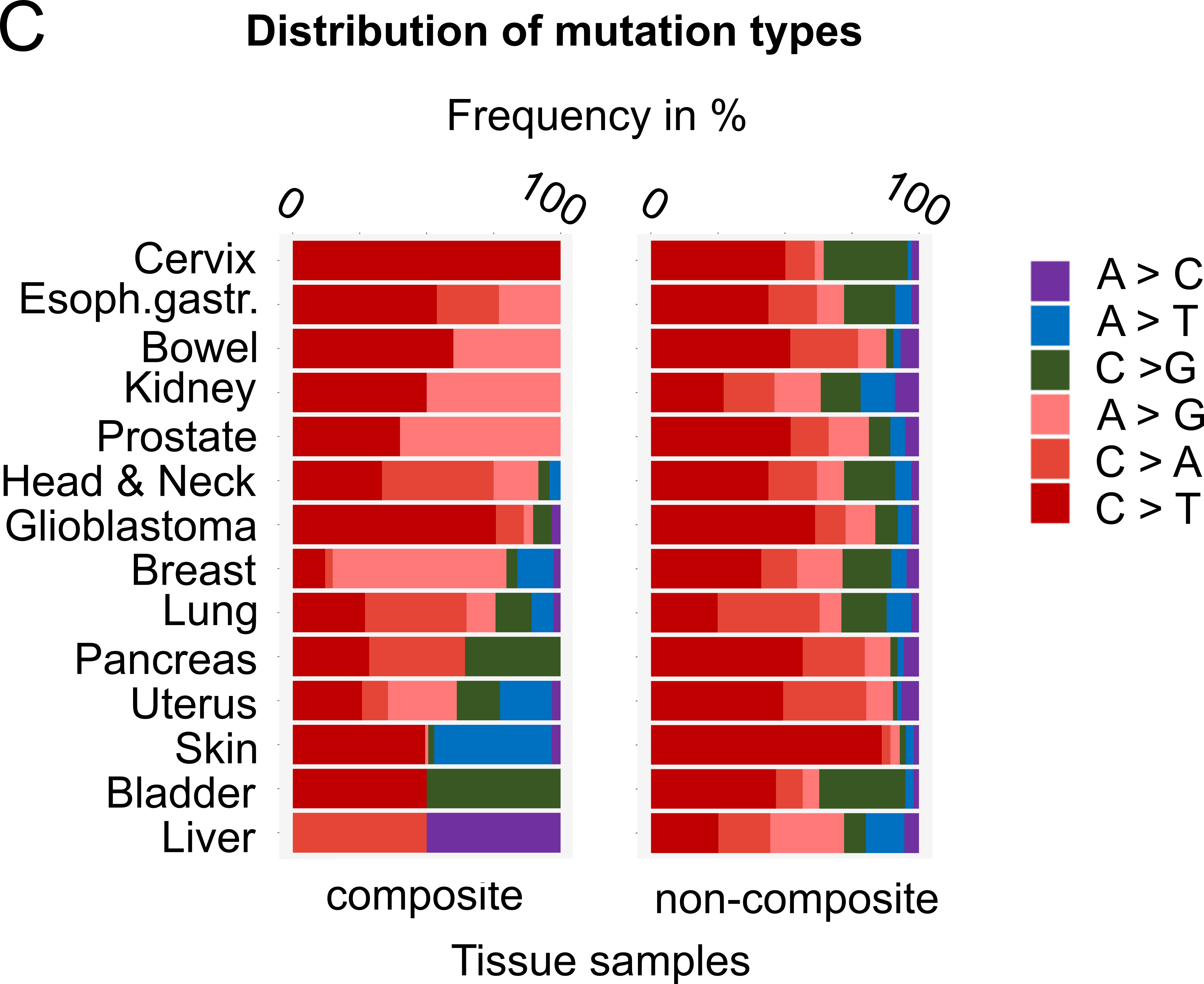


***Supplementary Figure 1 Overview of copy number variation and mutation data of the datasets, individual composite mutations and mutation signatures of composite mutations as analyzed.*** *a, The distribution of different mutations per tumor type show that many cases have either a gain (light red) or deletion (light blue). However, the distribution between point mutations, amplifications, and deep deletions are similar for all tumor types, which are the data we used for all the analyses. b, An lollipop overview of all composite mutations that occur for each gene, all these mutations are hotspot mutations. C, For each tumor type, plotted the distribution of nucleic acid changes of all mutations, for cases with (left) and without (right) composite mutations. The mutation signature for cases with composite mutations are similar to those commonly seen for replication errors and/or mutagen induced mutations. No clear difference was observed when compared to the non-composite mutated tumors.*

## Supplementary Figure 2


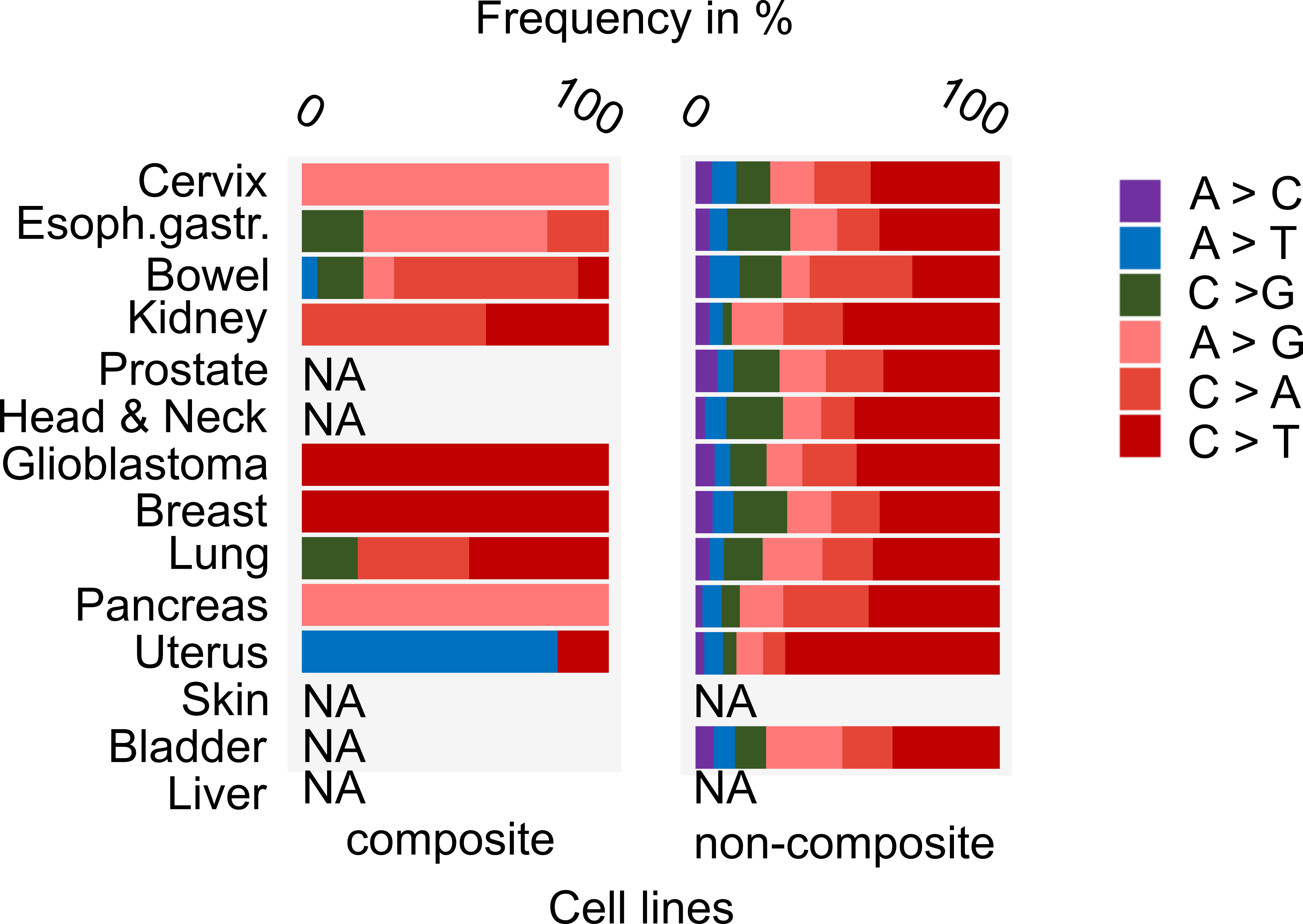


***Supplementary Figure 2 Mutation signature analysis on the cell line data did not show a clear difference between composite mutations and other mutations.*** *There are many missing values, due to the low sample size. However, most composite mutations are similar to those seen for replication errors and/or mutagen induced mutations (C>T mutation). No clear difference was observed when compared to the non-composite mutated tumors.*

## Supplementary Figure 3


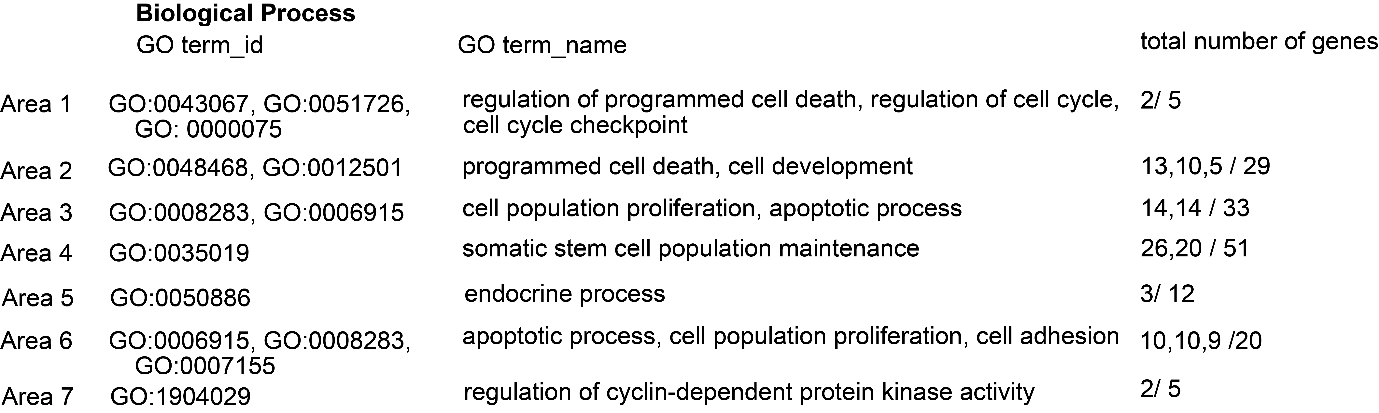


***Supplementary Figure 3 Composite mutations correlate with common patterns of co-mutations.*** *On the clustered areas of co-mutations per tumor type, we performed a gene ontology analysis. These areas resulted in pathways that are involved in cell cycle-related processes.*

## Supplementary Figure 4


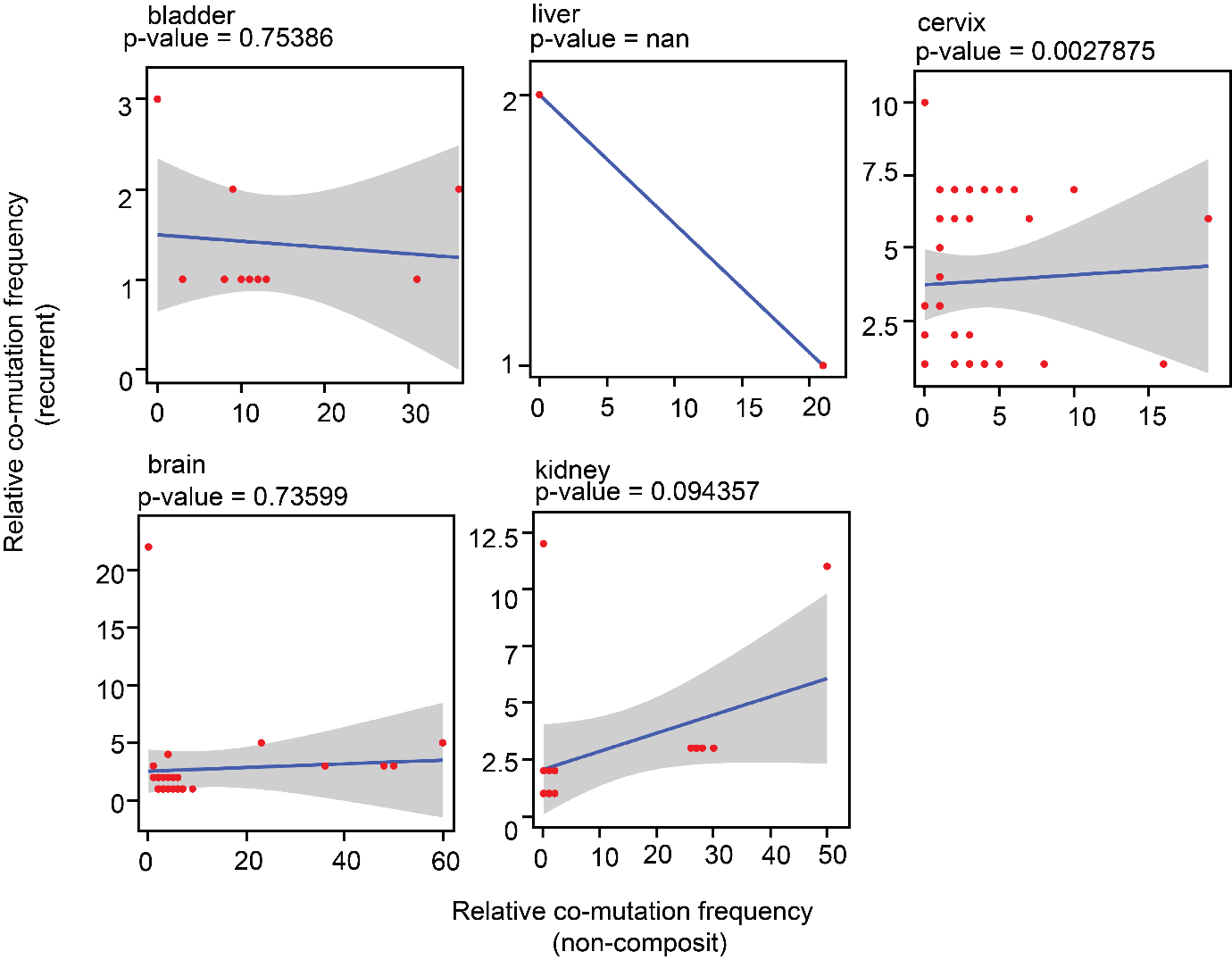


***Supplementary Figure 4 The heterogeneity in co-mutation profiles shows a complementary relationship.*** *To calculate the correlation between co-mutations seen in cases with composite mutations (y-axis) and cases without composite mutations (x-axis) we firstly plotted both. Subsequently, we calculated the slope and the p-value based on the direction of the variables of x and y. Here we show cases without a significant relation of frequencies of co-mutations that are found together with composite mutations versus frequencies seen in the absence of composite mutations.*

Supplementary Figure 5
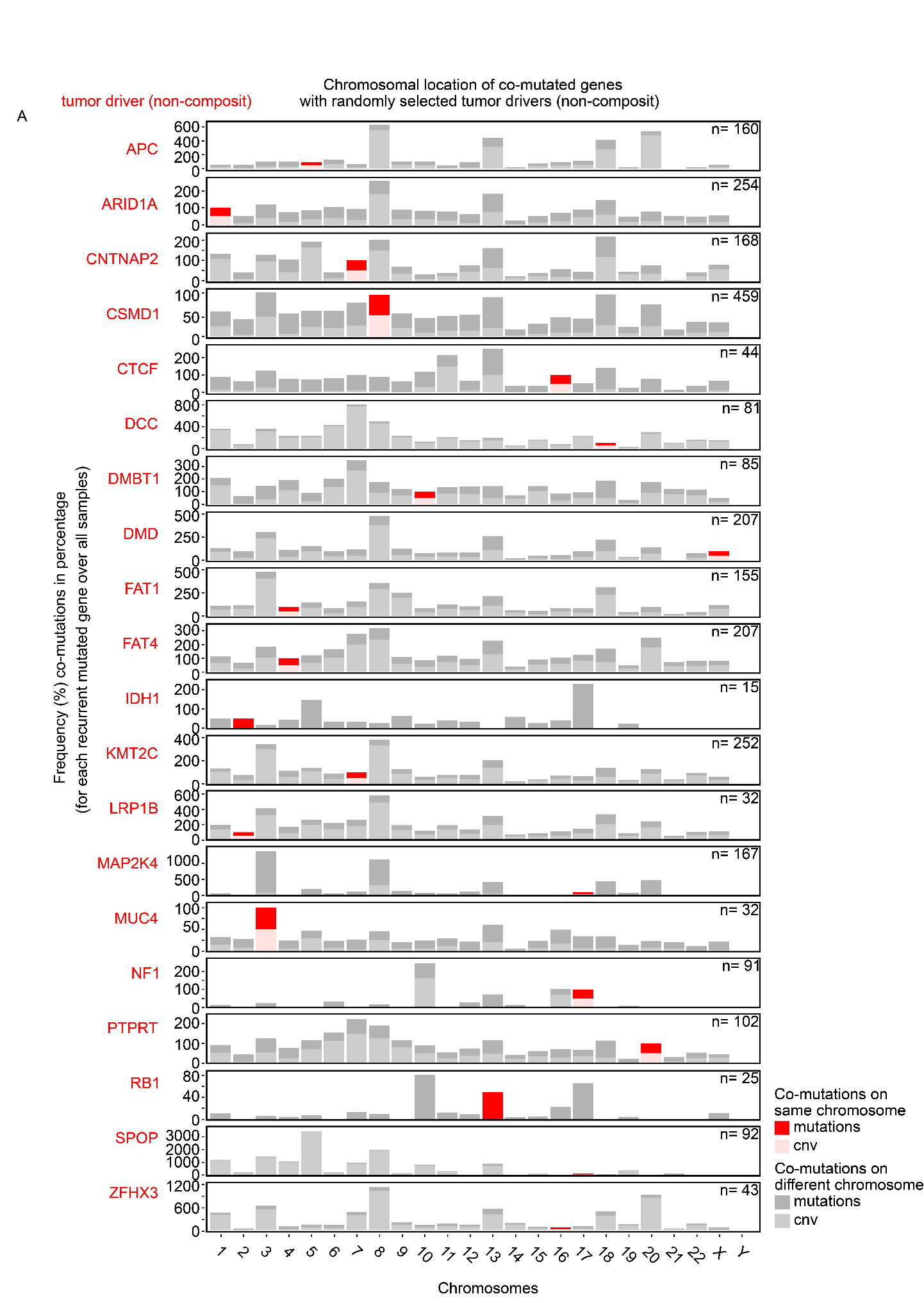


### (continued)


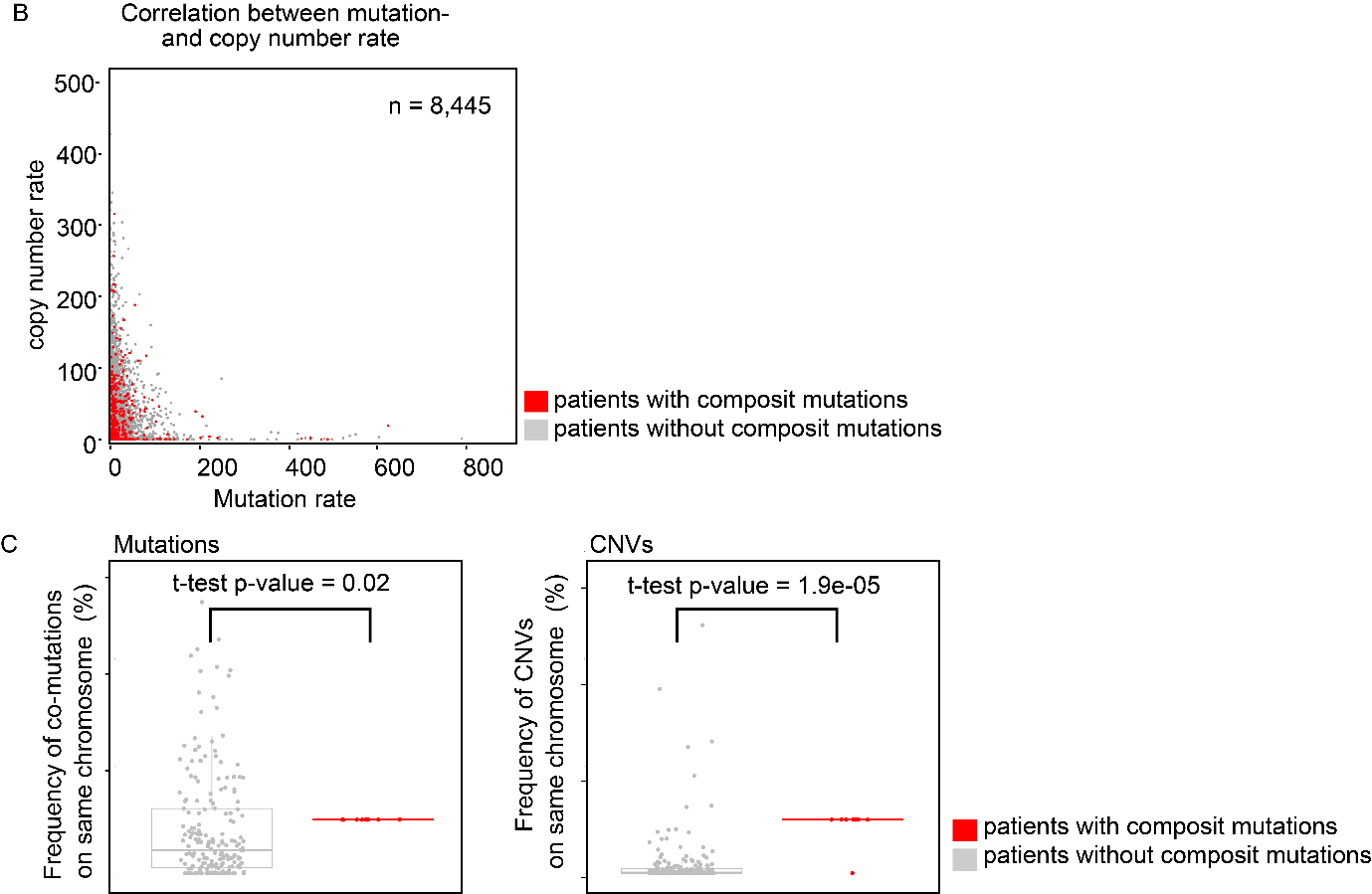


***Supplementary Figure 5 Validation co-mutations are enriched within the chromosomal territory of genes with composite mutations (chromosysmos).*** *a, we randomly selected 20 genes and identified them as composite mutated genes and mapped all mutations based on the chromosomal locations. Here we can not see an increase in co-mutations on the composite mutated gene (red). This validation confirms our finding that there is a hypermutation profile on a chromosomal area resulting in a second mutation in the same gene. b, From all the patients we potted the CNV rate towards the mutation rate and we can see no correlation. c, Comparing patients with composite mutations and without composite mutations, we can see a significant difference in mutations occurring on the composite mutated gene (p-value = 0.02 for mutations and p-value = 1.9e-05 for CNV).*

## Supplementary Figure 6


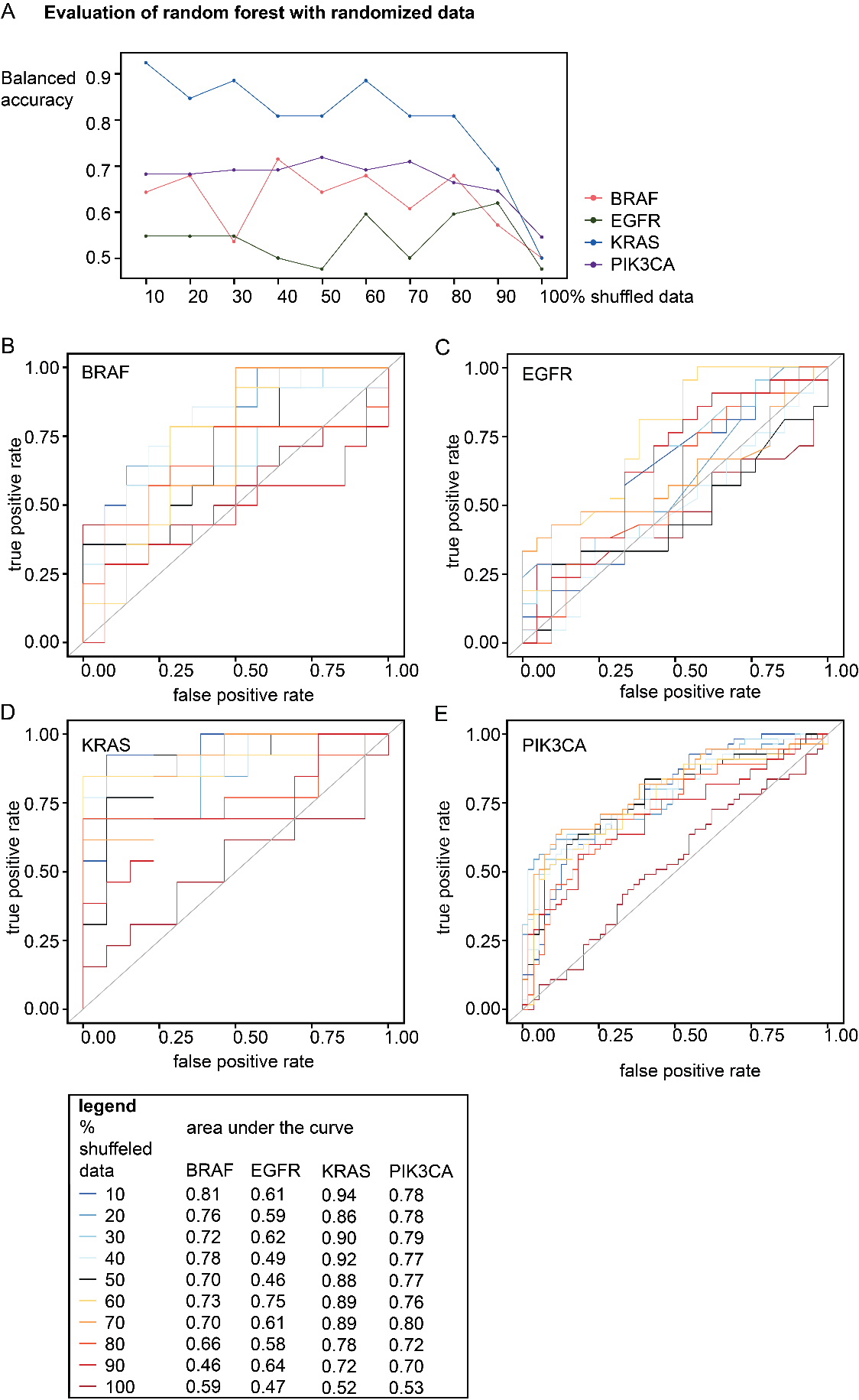


***Supplementary Figure 6 Evaluation of Random Forest model to predict composite mutations.*** *a, DNA mutation profiles of patients from tumor types that contain composite mutations in the genes BRAF, EGFR, KRAS, and PIK3CA were collected. All the features were shuffled in an increasing amount and the balanced accuracy was plotted. With an increasing amount of randomization, we can see the performance drop. b, c, d, e. The ROC curves show also a drop in performance when the data is randomized. When the full data is randomized we see that the performance is close to 0.5, meaning that the model makes random guesses.*

Supplementary Figure 7
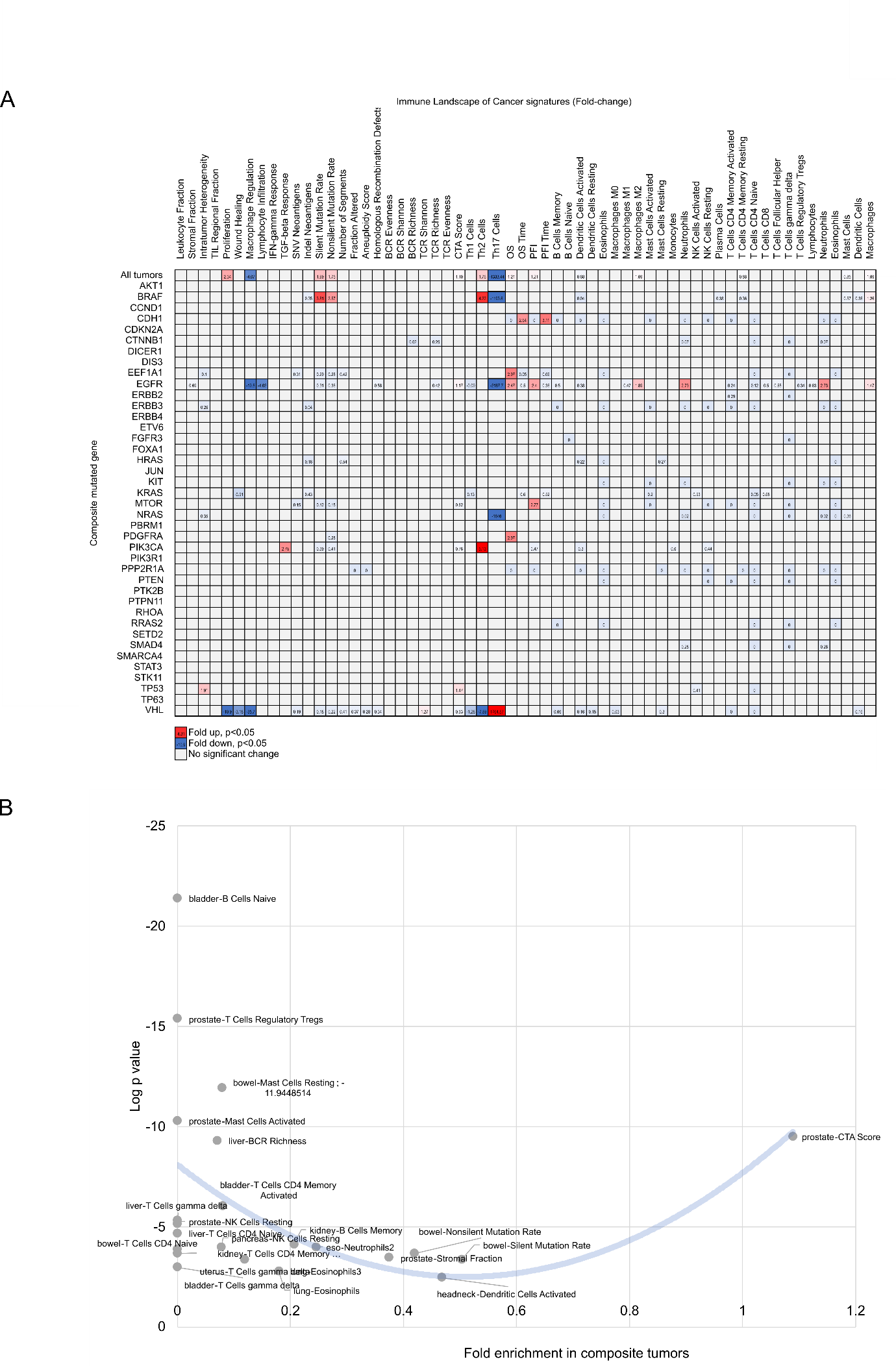


***Supplementary Figure 7. Composite mutations do not seem to be driven by the tumor microenvironment.*** *We analyzed whether composite mutations are correlated to genes expressed in the tumor microenvironment using gene signatures published by Thorsson et al^41^ using n=7,640 tumor specimens of which n=424 were composite mutations. We made comparisons between (A) composite mutation positive versus negative tumors; tumors with composite mutated genes versus negative tumors or (B) compared tissues with and without composite mutations. The data show only values in case the double sided t-test, corrected for multiple testing, was positive. The signatures fail to show a consistent pattern over the three comparisons. As an example, composite mutations associate with proliferation when all tumors are considered but fail to show this for individual genes. Many negative associations seem to be caused by stochastic events due to an unbalanced absence of signature-values rather than a consistent pattern and these are therefore considered false negative.*
